# Genetic and biochemical diversity of terpene biosynthesis in cyanobacterial strains from tropical soda lakes

**DOI:** 10.3389/fmicb.2025.1582103

**Published:** 2025-07-04

**Authors:** Mauricio J. Machado, Fernanda R. Jacinavicius, Rhuana V. Médice, Rafael B. Dextro, Anderson M. T. Feitosa, Marcio B. Weiss, Thierry A. Pellegrinetti, Simone R. Cotta, Camila M. Crnkovic, Marli F. Fiore

**Affiliations:** ^1^Center for Nuclear Energy in Agriculture, University of São Paulo, Piracicaba, Brazil; ^2^School of Pharmaceutical Sciences, University of São Paulo, São Paulo, Brazil; ^3^Faculty of Agricultural Sciences and Food, Université Laval, Québec City, QB, Canada; ^4^“Luiz de Queiroz” College of Agriculture, University of São Paulo, Piracicaba, Brazil

**Keywords:** biosynthetic gene cluster, carotenoids, environmental adaptation, genomic, isoprene, metabolomic

## Abstract

**Introduction:**

Terpenes and terpenoids are vital components in diverse metabolic pathways, forming the terpenome—the complete spectrum of terpene-related compounds biosynthesized by an organism. Integrating bioinformatic tools has significantly enhanced the ability to assess metabolic potential by combining these computational approaches with experimental biochemical data. Furthermore, gene annotation provides critical insights into specialized targets, facilitating the identification of shared or unique features across different strains.

**Aims and methods:**

This study investigates the presence of terpene compounds in cyanobacterial strains isolated from tropical soda lakes using a combination of gene mining, synteny analysis, phylogenetics, and metabolomics.

**Results and discussion:**

Key enzymes, including phytoene synthase and squalene hopene cyclase, were identified, showing significant similarities and evolutionary links to gene copies in Cyanobacteria from diverse ecological environments. Metabolomic analysis complemented genomic predictions, uncovering a rich diversity of tetraterpene compounds, particularly carotenoids. Notably, triterpene hopanoids were found exclusively in a unicellular strain. These compounds show significant potential for cellular protection, metabolic adaptation, and biotechnological uses. They might support microbial communities in extreme environments, such as the saline-alkaline lakes of the Pantanal Biome in Brazil, developing unique survival and resilience strategies in these harsh conditions.

**Conclusion:**

This study highlights the extensive range of insights that can be obtained by integrating genetics and biochemistry in exploring cyanobacterial diversity, especially from organisms thriving in extreme environments.

## 1 Introduction

The tropical soda lakes of Nhecolândia, Mato Grosso do Sul (MS), Brazil, are unique ecosystems of remarkable biological and ecological importance. These lakes in the Brazilian Pantanal biome are characterized by saline-alkaline conditions with a pH gradient varying between 8.62 and 10.26, salinity from 0.41 to 2.42 g L^–1^, and low water column level (< 2 m) (Pellegrinetti et al., 2023). The unique combination of high pH, salinity, and intense UV radiation in these lakes creates an environment that supports a distinctive microbial community, with cyanobacteria playing a crucial role in sustaining its diversity (Cotta et al., 2022). Specifically, the genera *Limnospira* and *Anabaenopsis* dominate in these lakes, creating blooms that enhance the environment’s unique characteristics and support a diversity of heterotrophic organisms that depend on or are modulated by their presence (Andreote et al., 2018; Pellegrinetti et al., 2024). The adaptability of cyanobacteria to these adverse environmental conditions is truly remarkable (Waditee-Sirisattha and Kageyama, 2022; Jacinavicius et al., 2021). The ability of cyanobacteria to adapt and thrive in various ecological niches reflects their metabolic versatility. This phylum possesses a vast diversity of specialized metabolites, such as alkaloids, amino acid derivatives, fatty acid derivatives, polyketides, peptides, and terpenes (Chlipala et al., 2011; Jones et al., 2021; Weiss et al., 2025).

Terpenoid compounds are a diverse group of natural products with more than 80,000 representatives in plants, fungi, marine invertebrates, and bacteria (Christianson, 2017; Rudolf et al., 2021). The terpenome embraces all compounds that contain isoprenoids produced from building blocks of dimethylallyl-pyrophosphate (DMAPP) and isopentenyl-pyrophosphate (IPP) that can be further converted into different molecules by terpene synthases (TPS). Terpene synthases are responsible for the structural diversity found in the terpenoid natural products. Terpenoids are classified based on the number of carbon atoms (C) in their core structure: hemiterpenes (C5), monoterpenes (C10), sesquiterpenes (C15), diterpenes (C20), triterpenes (C30), and tetraterpenes (C40). Additionally, some meroterpenes have been identified, which are hybrid molecules resulting from mixed biosynthesis (Nazir et al., 2021; Rudolf et al., 2021). Natural products such as steroids, vitamins, plant hormones, and drugs highly utilized in medicine (e.g., taxol and artemisinin) represent terpene compounds (Tholl, 2006; Avalos et al., 2022). Terpenoids are essential in the cellular metabolism of photosynthetic organisms, acting in light conversion, membrane fluidity, and assemblage of photosynthetic reaction centers. In oxygenic phototrophic bacteria, such as cyanobacteria, isoprenoids are synthesized from the methylerythritol-phosphate (MEP) pathway, which relies on glyceraldehyde-3-phosphate (G3P) and pyruvate, both derived from photosynthesis (Pattanaik and Lindberg, 2015).

The carbon metabolism of cyanobacteria, which can convert CO_2_ into a wide range of terpenoid compounds, makes these microorganisms an emerging target host for biochemical production, and thus a promising avenue for future research. However, challenges need to be overcome, like carbon partitioning and balancing pigment production (Lin and Pakrasi, 2019). Specifically looking at terpenoids, cyanobacteria have a large diversity of these compounds beyond only pigments, with a considerable variation of functions and structures, which have been considered important sources of molecules to produce medicines, biofuels, and other applications (Nandagopal et al., 2021). Among the sesquiterpenes that can be engineered in cyanobacteria, farnesene is a compound that has many applications in different areas like biofuels, pest management, cosmetics, flavors, and fragrances (Rautela et al., 2024). The diterpenoid compounds tolypodiol and noscomin, isolated from *Tolypothrix nodosa* and *Nostoc commune*, respectively, exhibit significant anti-inflammatory and antimicrobial activity (Prinsep et al., 1996; Jaki et al., 1999). Additionally, the sesterterpenes cybastacines A and B, derived from *Nostoc* sp., and scytoscalarol from *Scytonema* sp., also possess noteworthy antimicrobial effects (Mo et al., 2009; Cabanillas et al., 2018). Also, cyanobacterial tetraterpenes can act as an antioxidant agent, principally reducing reactive oxygen species (ROS) and in therapeutic solutions for inflammation-related skin disorders (Hatha and Sumayya, 2023; Morone et al., 2024).

Abiotic stress is known to alter products of the isoprenoid pathway; however, its specific effects on cyanobacterial metabolism remain underexplored. In response to the extreme conditions of soda lakes, such as high pH, salinity, and UV exposure, Cyanobacteria, which dominate in these environments, exhibit specific metabolic pathways that help them adapt and thrive. The increasing availability of genomic data and advancements in metabolomics have significantly boosted the discovery of specialized metabolites, providing new sources for identifying and characterizing cyanobacterial compounds (Winter et al., 2011; Micallef et al., 2015; Weiss et al., 2023). However, further research into cyanobacterial metabolism is essential for a comprehensive understanding of these metabolic adaptations and bioprospecting natural products from organisms isolated from extreme environments.

This study aimed to characterize terpene and terpenoid gene clusters in the genomes of cyanobacterial strains isolated from tropical soda lakes in the Brazilian Pantanal, considered extreme environments. Additionally, it evaluated the biosynthesis of terpene compounds and their potential biological roles, providing insights into the metabolic adaptations of microorganisms to harsh conditions and underscoring the bioprospecting potential of cyanobacterial metabolites.

## 2 Materials and methods

### 2.1 Cyanobacteria culture conditions and metabolites extraction

The cyanobacterial strains, *Anabaenopsis elenkinii* CCIBt3563, *Pantanalinema rosaneae* CENA516, *Geminocystis* sp. CENA526, *Alkalinema pantanalense* CENA528, *Limnospira platensis* CENA597, and *Limnospira platensis* CENA650, isolated from soda lakes in the Brazilian Pantanal biome, were used in this study and maintained as uni-cyanobacterial cultures (Supplementary Table 1). These strains are part of the culture collections of the Cell and Molecular Biology Laboratory (CENA/USP) in Piracicaba, São Paulo, Brazil, and the Cyanobacterial Culture Collection (CCIBt) at the Institute of Botany in São Paulo, São Paulo, Brazil. The cyanobacterial strains were cultured in Z8 medium (Kotai, 1972) or a modified Z8 medium containing 7.5 g. L^–1^ of NaCl and adjusted to pH 9.5. Cultures were maintained at 22 ± 1°C under a 14/10 h light/dark photoperiod with fluorescent illumination (40 μMol photon m^–2^.s^–1^). After 30 days of growth, the culture biomass was lyophilized and stored at −20°C for subsequent extraction.

Freeze-dried cyanobacterial biomass was extracted using three different solvent systems—methanol/water (1:1, v/v), ethyl acetate, and dichloromethane/methanol (1:1, v/v)—applied independently to separate aliquots of the same biomass at a 10:1 (v/w) solvent-to-biomass ratio. Extracts were sonicated (Ultrasonic cleaner, Unique) for 1 min and kept overnight at room temperature for complete extraction. Afterward, extracts were filtered through qualitative paper filters (80 g.m^–2^), and all filtrates were evaporated overnight under nitrogen flow. Dried extracts were resuspended in methanol (10 mg.mL^–1^) and subjected to a clean-up step using SPE Columns C18 (Applied Separations).

### 2.2 DNA extraction and genome sequencing

After 30 days of cultivation under the described conditions, cultures of *P. rosaneae* CENA516; *Geminocystis* sp. CENA526; *A. pantanalense* CENA528; *L. platensis* CENA597 and *L. platensis* CENA650 were concentrated by centrifugation at 5,000 × *g* for 10 min. The concentrated biomass was used for total DNA extraction using the AxyPrep Bacterial Genomic DNA Miniprep Kit (Axygen Biosciences, Union City, CA, United States) according to the manufacturer’s requirements. The quality of the DNA was confirmed using 1% (m/w) of agarose gel. DNA quantification was performed with a Qubit 2.0 Fluorometer, using a Qubit dsDNA BR Assay Kit (Life Technologies, Carlsbad, CA, United States). An amount of 1 μg of DNA was used to prepare paired-ends libraries with the Kit Nextera DNA Flex (Illumina, San Diego, CA, United States), which was sequenced in a platform HiSeq 2500 (Illumina) following the manufacturer’s instructions. The gDNA was then sent to the Joint Genome Institute (JGI) (Project ID: 504264), where a PacBio SMRTbell library was prepared for circular consensus sequencing (CCS) and sequenced using the PacBio HiFi platform.

### 2.3 Genome assembly and bioinformatics analysis

The quality of the reads obtained from genomic sequencing on the HiSeq and HiFi platforms was evaluated using FastQC v0.11.8 (FastQC), with analysis graphs generated to assess the quality metrics. Adapter sequences were subsequently removed using NxTrim v0.4.3 (O’Connell et al., 2015). Further filtering of reads was performed with Cutadapt v1.18 (Martin, 2011), which excluded sequences with phred quality scores below 30, lengths shorter than 30 bp, and repetitive elements. The resulting high-quality short reads were individually assembled using SPAdes v3.13.0 (Bankevich et al., 2012) with the metaSPAdes module for metagenomic assembly (Nurk et al., 2017), following the methodology described by Alvarenga et al., 2017. Long-read sequences were assembled using Flye v2.9.1 (Kolmogorov et al., 2019), and hybrid assemblies, which combined both types of data, were generated using SPAdes in hybrid mode. Taxonomic classification of assemblies was performed using Kraken2 (Wood et al., 2019), while contaminant sequences were identified and removed using Krakentools (Lu et al., 2022). Pilon (Walker et al., 2014) was employed for genome polishing, correcting assembly errors, and, along with the programs SSPACE (Boetzer et al., 2011) and GAPPadder (Chu et al., 2019), was used for closing sequence gaps, thereby enhancing the completeness and accuracy of the final genomes. The assembled genomes were assessed for quality and completeness using QUAST v5.0.2 (Gurevich et al., 2013). Genome integrity and contamination were estimated using CheckM v1.0.13 (Parks et al., 2015). Additionally, BUSCO v3.0 (Simão et al., 2015) was utilized to evaluate genome quality by identifying conserved single-copy orthologs. The genomic data for *A. elenkinii* can be accessed through the NCBI GenBank database (National Center for Biotechnology Information) under the accession number CP063311. The assembled genomes studied are also deposited in NCBI (BioProject number: PRJNA1226917).

Genome annotation was performed with a rapid prokaryotic sequence annotation algorithm implemented in Prokka v1.12 (Seemann, 2014). The presence of genes associated with the methylerythritol phosphate (MEP) pathway, plus other terpenoid biosynthetic pathways, such as triterpenes, tetraterpenes, and meroterpenoids, were identified through manual curation and using BlastKOALA tool (Kanehisa et al., 2016). Biosynthetic Gene Clusters (BCGs) related to terpene production were predicted by submitting the genome assemblies to AntiSMASH bacterial version 6.0 (Blin et al., 2021) using default parameters. For insights into their evolution and potential differences in the pathways they encode, the predicted BCGs were analyzed with Clinker to visualize and compare gene cluster architectures across the strains using default settings (Gilchrist and Chooi, 2021). Additionally, orthologous gene clusters among the genomes were compared using OrthoVenn2, allowing for the identification of shared and unique genes across the studied strains (Xu et al., 2019).

### 2.4 Gene phylogeny analysis

Amino acid sequences of the genes encoding phytoene synthase (*crt*B) and terpene synthase (*sqh*C) annotated on the Pantanal strains’ genomes were compared to the National Center for Biotechnology Information (NCBI) database via BLAST. Homologs were selected based on coverage (> 95%) and identity scores (> 70%, *e*-value > 1.10–50). Due to the high variability of terpene synthases, genes associated with the biosynthesis of geosmin and germacrene were included in the analysis to enhance resolution and more effectively explore evolutionary relationships. A maximum-likelihood phylogenetic analysis was constructed for both enzymes in MEGA11 (Tamura et al., 2021) using the substitution model Jones-Taylor-Thornton (JTT) based on matrixes of differential substitution rate for each amino acid from the protein sequence. For robustness, 1,000 bootstrap replication steps were used.

### 2.5 HPLC-MS/MS analysis

The HPLC-MS/MS analyses were carried out on a high-performance liquid chromatography system (Shimadzu© Prominence Liquid Chromatography) coupled with a high-resolution tandem mass spectrometer (Micro TOF-QII; Bruker Daltonics©, MA, United States) with electrospray ionization source (ESI)—(HPLC-ESI-QTOF-MS/MS). Chromatographic separations were performed over a Kinetex C18 column (2.1 × 50 mm × 1.7 μM, Phenomenex) equipped with pre-column. Sample injection volumes were 2 μL and the mobile phase consisted of 0.1% v/v formic acid in ultrapure water (solvent A) and 0.1% v/v formic acid in acetonitrile (solvent B). The gradient was as follows: 10–100% B (7 min), 100% B (in 1 min), 100–10% B (0.1 min), 10% B (in 1.9 min). Flow rate was 0.5 mL/min. The ESI conditions were capillary potential at 3.5 kV, drying gas (N_2_) at 200°C at a flow rate of 9.0 mL/min, and nebulization pressure at 43.0 psi. The mass spectrometer (QTOF) was operated in auto-scan MS/MS mode, and mass spectra were acquired in positive mode, with collision-induced dissociation (CID) energy at 60 eV (isolation mass of 100 *m/z* with width of 3 *m/z*, and isolation of mass 500 *m/z* with width of 6 *m/z*) or 70 eV (isolation mass of 1,000 *m/z* with width of 9 *m/z*, and isolation of mass 1,500 *m/z* with width of 12 *m/z*) and averaged stepping of 65–100% CID. The mass spectra were acquired in the mass range of 150–2,000 Da (MS1) and 50–2,000 Da (MS2).

### 2.6 Terpenoid profile investigation

LC-MS/MS data were converted to “.mzXML” format in MSConvert© software from ProteoWizard tools. Data was filtered by removing MS/MS fragment ions within ± 17 Da of the precursor *m/z*. Window filtering selected the top 6 fragment ions in a ± 50 Da window. The converted data were processed using the Classical Molecular Networking pipeline of the GNPS platform^1^ (Wang et al., 2016). The precursor ion mass tolerance was 0.02 Da, and the MS/MS fragment ion tolerance was 0.02 Da. The minimum cosine score was 0.7, and at least 6 matched peaks. Network spectra were compared against GNPS’ spectral libraries, with matches requiring a score above 0.7 and at least 6 matched peaks. Additional filters included precursor window settings and exclusion of spectra identified as blanks before networking. Cytoscape© software was used to visualize molecular networks. *In silico* structure annotations from the GNPS library were incorporated to enhance the chemical structural information within the molecular network. *In silico* structure annotations were incorporated using GNPS tools such as Dereplicator, Dereplicator+, and the *Insilico* Peptidic Natural Product Dereplicator (Gurevich et al., 2018; Mohimani et al., 2018), enhancing identification of peptidic and non-peptidic natural products. The search results were integrated into the GNPS MolNetEnhancer workflow, enabling annotation of a wide range of chemical classes, including lipids and lipid-like molecules, alkaloids, phenolic compounds, terpenoids (monoterpenes, sesquiterpenes, and carotenoids), and peptides (Ernst et al., 2019). Chemical class annotations were performed using the ClassyFire chemical ontology. For putative metabolite identification, HRMS data were also processed using DataAnalysis 4.4 and MetaboScape 4.0 software (Bruker Daltonics, Germany). Accurate masses were used to search against CyanoMetDB (Jones et al., 2021), PubChem, ChemSpider, METLIN (Smith et al., 2005), NPAtlas (Van Santen et al., 2019), the Dictionary of Natural Products, Bruker’s MetaboBASE Plant Library, MetaboBASE Personal Library 3.0, and in-house databases. Additional database searching was performed using GNPS Theoretical/*In silico* tools, SIRIUS 4, and CANOPUS (Duhrkop et al., 2019, 2021). Elementary compositions and deviations from theoretical values (ppm error) were calculated using the SmartFormula algorithm, adopting a 5 ppm threshold. Natural isotopic patterns were considered to refine formula predictions and to aid in matching experimental MS/MS spectra with published data. Furthermore, the ChemCalc web service^2^ (Patiny and Borel, 2013) was used for additional molecular formula predictions and mass error calculation, and the Carotenoids Database^3^ (Yabuzaki, 2017) was used for prospecting carotenoids.

## 3 Results

### 3.1 Genomics analysis

*Anabaenopsis elenkinii* CCIBt3563, *Pantanalinema rosaneae* CENA516, *Geminocystis* sp. CENA526, *Alkalinema pantanalense* CENA528, *Limnospira platensis* CENA597, and *Limnospira platensis* CENA650 had a complete set of MEP pathway genes responsible for IPP and DMAPP biosynthesis (Supplementary Figure 1). Among these coding regions, genes associated with the MEP pathway of terpene biosynthesis (*dxr*, *dxs*, *isp*D, *isp*E, *isp*F, *isp*G, *isp*H, and *idi*) were orthologous to all evaluated strains from the Pantanal saline-alkaline lakes. The targeted genes are scattered in the genomes rather than organized in clusters. Gene size and identity were conserved among the different sequences from the six Pantanal cyanobacteria that were evaluated. Orthologous gene analysis of the complete genomes highlighted that amongst 9,537 proteins predicted in the six genomes evaluated, 1,507 proteins formed common clusters (Supplementary Figure 2); among them, several genes related to terpene biosynthesis were identified, mainly related to the carotenoid pigments.

**FIGURE 1:**
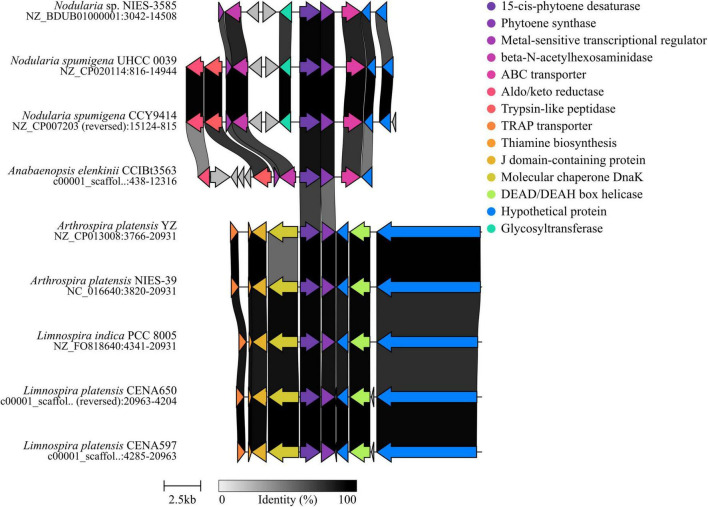
Synteny analysis of phytoene synthase gene clusters predicted by the AntiSMASH platform revealed high similarity to the biosynthetic gene cluster (BGC) in *A. elenkinii* CIBt3563, *L. platensis* CENA597, and CENA650. The genes encoding phytoene synthase (*crt*B) and 15-*cis*-phytoene synthase (*crt*P) were consistently located at central positions within the analyzed sequences.

Analysis using AntiSMASH predicted two secondary metabolite biosynthetic gene clusters (BGCs) with significant identity (> 50%) for terpenes among the Pantanal cyanobacterial genomes. The first was for scalene-hopene cyclase, and the second was for phytoene synthase. The scalene hopene cyclase (SCH) enzyme, encoded by the gene *sqh*C, is linked with the cyclization catalysis of hopanoids from the acyclic precursor squalene. This gene is associated with the precursor of triterpenoid compounds (hopene and hopanol), which BlastKOALA predicted in the strains *P. rosaneae* CENA516, *Geminocystis* sp. CENA526, and *A. pantanalense* CENA528 (Supplementary Table 2). The SHC enzyme clusters predicted by AntiSMASH were compared to data available in the platform’s database. Synteny analysis for the scalene hopene cyclase gene found several homologs from different genera and two other strains of *Geminocystis* with significant sequence similarity compared to the Pantanal strains’ genomes (Supplementary Figure 3). Similarly, genes related to phytoene synthase (CrtB), the first enzyme on the carotenoid biosynthetic pathway, were also predicted by AntiSMASH. The studied genomes showed the presence of genes related to phytoene synthase (*crt*B) and 15-sis-phytoene-desaturase (*crt*P). The *crt*B gene clusters found in *A. elenkinii* CCIBt3563 and both strains of *L. platensis* (CENA597 and CENA650) showed the highest similarity to strains of *Nodularia* and the *Arthrospira* genus found in the database that presented this same gene concatenated in the genome (Figure 1). Notably, the strains of *Nodularia* sp. NIES-3585 and *Nodularia spumigena* (UHCC 0039 and CCY 9414), exhibiting significant similarity, are sourced from marine environments, while the strains related to *L. platensis* are from freshwater habitats. The similarity of the *crt*B gene in phylogenetically related strains from distinct aquatic environments suggests that the carotenoid biosynthesis is conserved among the same cyanobacteria species inhabiting different ecological niches.

As for the phylogenetic analysis, the *crt*B gene tree (Supplementary Figure 4) showed that the gene copies found in the strains from saline lakes in Pantanal may exhibit different evolutionary trajectories, presenting common ancestry among the close phylogenetic members. The genes identified in the *L. platensis* strains (CENA597 and CENA650) grouped with homologs retrieved from other *Arthrospira* and *Limnospira* strains, forming a basal group on the tree, with an early ancestry to the remaining copies of phytoene synthase used in the analysis. Meanwhile, *crt*B annotated in *A. elenkinii* CCIBt3563 clusters with other *Anabaenopsis* strains, sharing a common ancestry with *Nodularia* strains and *crt*B genes from various distinct genera. While the *A. pantanalense* CENA528 gene appears to be ancestral to two others freshwater *Alkalinema* strains, the close branch formed by the MAG of *Alkalinema* sp. CACIAM 70d represents an environmental sample isolated from the Amazon biome in Brazil. Furthermore, the strains *P. rosanae* CENA516 and *Geminocystis* sp. CENA526 clustered together, sharing a common ancestor with the strain *Pantanalinema* sp. GBBB05, which was isolated from a freshwater environment in the Cerrado biome, also in Brazil. As expected, the *sqh*C gene phylogenetic tree (Supplementary Figure 5) displays an external group formed by terpene synthases of *Nostoc* strains associated with the sesquiterpenes compounds geosmin, selinene, and germacrene. The addition of other terpene synthases aimed to capture a broader range of biosynthetic diversity and provide deeper insights into terpene production’s evolutionary patterns. This allowed the grouping of all squalene/hopene cyclases with a common ancestral state. *Geminocystis* sp. CENA526 is grouped with other *Gemnocystis* sp. and a *Cyanobacterium aponinum* strain, both of which are from freshwater, forming the sister branch to all remaining copies of the *sqh*C gene. Both *P. rosaneae* CENA516 and *A. pantanalense* CENA528 shared ancestry with *Synechococcus and Thermosynechococcus* strains. The tree presented in this work appears to follow the same topology as 16S rRNA gene classification, indicating that this gene could be related to the phylogeny of the strains.

Genes related to tetraterpene and meroterpene biosynthetic pathways were also annotated in the genomes and grouped as orthologs (Supplementary Table 2). The tetraterpene group, composed of carotenoids, was predicted on the genomes by BlastKOALA and showed variations among the genomes analyzed (Supplementary Table 2). All strains presented genes corresponding to carotenoid biosynthesis (*crt*B, *crt*P, *crt*Q, *crt*H, *crt*R), with genes related to the production of lycopene, beta-carotene, myxol group compounds, beta-cryptoxanthin, zeaxanthin, and hydroxyechinenone. However, only *P. rosaneae* CENA516, *Geminocystis* sp. CENA526 and *A. pantanalense* CENA528 possess the *crt*S, *crt*W, and *crt*X genes, which are essential for the biosynthesis of various carotenoids, including echinenone, canthaxanthin, (3S,2′S)-4-ketomyxol-2′-alpha-L-fucoside, hydroxyequinenone, phenicoxanthin, adonixanthin, astaxanthin, and zeaxanthin diglucoside. Also, *Geminocystis* sp. CENA526, *L. platensis* CENA597, *L. platensis* CENA650, and *A. elenkinii* CCIBt3563 presented gene *crt*U (carotenoid phi/chi ring synthase) associated with the synthesis of the pigment isorenieratene.

The meroterpene group, characterized by its mixed biosynthetic pathways, was annotated across all examined strains. Specifically, the genes *ubi*A (4-hydroxybenzoate polyprenyltransferase), which is involved in the synthesis of ubiquinone, and *men*A (2-carboxy-1,4-naphthoquinone phytyltransferase), a key component of the phylloquinol (vitamin K) cycle, were annotated. Furthermore, were identified the genes *hpt* (homogentisate phytyltransferase) and *hggt* (homogentisate geranylgeranyltransferase) that play critical roles in the vitamin E biosynthetic pathway (Supplementary Table 2).

### 3.2 Terpenoid annotation and biosynthesis

**FIGURE 2:**
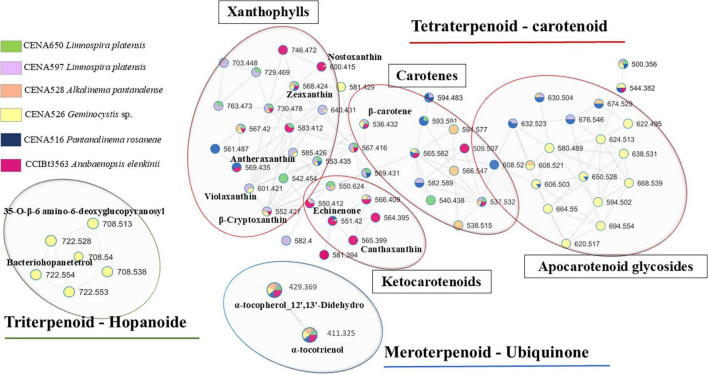
Molecular network of different extracts produced by cyanobacteria from tropical soda lakes. Nodes display the observed *m/z* values (parent ion) and their putative annotation; nodes were color-coded according to the source strains. Only clusters representing terpenoids are shown.

Many terpenoids were annotated in the cyanobacterial strains *A. elenkinii* CCIBt3563, *P. rosaneae* CENA516, *Geminocystis* sp. CENA526, *A. pantanalense* CENA528, *L. platensis* CENA597 and CENA650. They were grouped into three terpene classes (triterpenes, tetraterpenes, and meroterpenes) through molecular networking and by comparing experimental spectra with the GNPS database (Figure 2). The difference between the theoretical mass and accurate mass values (calculated error Δ) for each compound and their MS/MS fragmentation profiles are presented in Supplementary Table 3 and Supplementary Figures 6–15.

**FIGURE 3:**
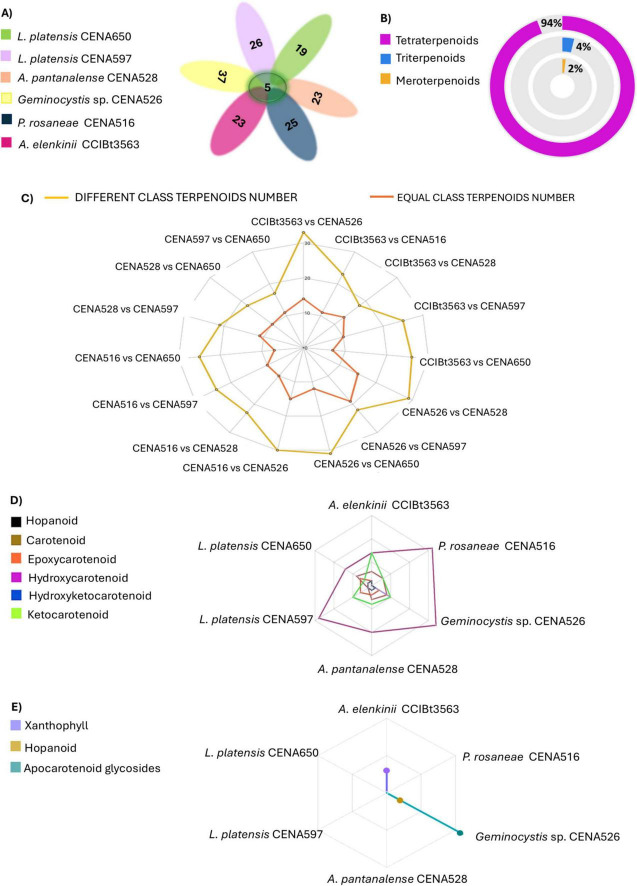
**(A)** Venn Diagram representing the number of nodes annotated as a terpenoid in each strain for terpenoid production and common to all samples. **(B)** Proportion of terpenoids found in cyanobacteria from tropical soda lakes. **(C)** Spider plot depicting the distribution of compound classes across different strains. **(D)** Spider plot showing the distribution of different groups of terpene compounds for each strain. **(E)** Spider plot presenting the exclusive presence of groups of terpene compounds in strains isolated from Pantanal.

The number and distribution of features related to terpenoid compounds across different cyanobacterial strains are shown in the Venn diagram (Figure 3A). This analysis revealed that most features represent tetraterpenoids, which comprise 94% of the total detected features, while triterpenoids and meroterpenoids comprise 4 and 2%, respectively (Figure 3B). Precursor genes related to α-tocopherol, α-tocotrienol (vitamin E), and phylloquinol (vitamin K) were found in the genomes of all strains (Supplementary Table 2). The compounds tocopherol (*m/z* 429.3708) and α-tocotrienol (*m/z* 411.3251) were annotated in MetaboScape and dereplicator GNPS (Figure 3 and Supplementary Tables 3, 4). *Geminocystis* sp. CENA526 stands out for its remarkable diversity of detected carotenoids, including unique compounds and genes for hopanoids, 35-O-β-6-amino-6-deoxyglucopyranosyl (*m/z* 708.5330 [M + H]^+^, C_41_H_73_NO_8_, Δ = −0.87 ppm) and bacteriohopanetetrol (*m/z* 722.5465 [M + H]^+^, C_42_H_75_NO_8_, Δ = −3.83 ppm), which are exclusive to this strain (Figure 3 and Supplementary Tables 2, 3).

The comparative analysis of terpenoid profiles among the Pantanal cyanobacterial strains revealed a significant insight, such as the observation of a greater dissimilarity of compounds rather than a homogenous profile amongst the studied cyanobacteria (Figure 3C). Strains CENA597 and CENA650 (*L. platensis*), belonging to the same species, showed a similarity of 39.29%. The highest similarity, at 46.34%, was between *Geminocystis* sp. CENA526 and *L. platensis* CENA597, despite these strains belonging to different families and having different cell morphologies (coccoid unicellular and filamentous, respectively). The lowest similarity was observed between *A. elenkinii* CCIBt3563 and *Geminocystis* sp. CENA526. Regarding chemical diversity and the predominance of specific terpenoids, it is noteworthy that hydroxycarotenoids were more prevalent in the strains *P. rosaneae* CENA516, *Geminocystis* sp. CENA526, *A. pantanalense* CENA528, and *L. platensis* CENA597 (Figure 3D). On the other hand, strain *A. elenkinii* CCIBt3563 showed more diversity across the categories (except hopanoids) and some exclusive xanthophyll compounds (Figure 3E and Supplementary Table 3). The terpenoid profiles varied among the studied strains. *P. rosaneae* CENA516, *Geminocystis* sp. CENA526 and *L. platensis* CENA597 stood out for their high concentration of hydroxycarotenoids, while *A. elenkinii* CCIBt3563 exhibited a relatively high diversity of ketocarotenoids (Figure 3C and Supplementary Table 2). *Geminocystis* sp. CENA526 exhibited a higher abundance and diverse terpenoid categories, including the exclusive presence of apocarotenoid glycosides and hopanoids (Figure 3E and Supplementary Table 2).

The analysis of carotenoids in cyanobacteria revealed that these compounds are predominantly represented by C40 cyclic species, which can be categorized into two major classes: carotenes (e.g., β-carotene) and xanthophylls (oxygenated derivatives of carotenes such as zeaxanthin and echinenone) (Supplementary Table 2). Based on the spectral similarity between samples and the GNPS database, the feature with *m/z* 565.4033 [M + H]^+^ was annotated as the terpene canthaxanthin with a calculated error of Δ = −2.29 ppm. This annotation was further supported by manual analysis of the isotopic and fragmentation patterns, with characteristic fragments (*m/z* 203.1, *m/z* 105.06, and *m/z* 119.08) of canthaxanthin. The SIRIUS platform also proposed the molecular formula C_40_H_52_O_2_ and classified the feature as a terpene. Similarly, the feature detected at *m/z* 551.4186 [M + H]^+^ was identified as echinenone (C_40_H_54_O, Δ = 2.05 ppm) based on MS data, which showed characteristic fragments (*m/z* 203.1, *m/z* 93.0, and *m/z* 69.0). The feature acquired with *m/z* 585.4231 [M + H]^+^ was suggested to be antheraxanthin (C_40_H_56_O_3_, Δ = 0.26 ppm), with fragments (*m/z* 95 and *m/z* 105) consistent with this carotenoid. These features were used as reference points for the annotation propagation of the other compounds in the network. Three compounds were consistently annotated among the analyzed strains: β-carotene, echinenone, and zeaxanthin (Figure 3 and Supplementary Table 4).

The biosynthesis of carotenoids begins with phytoene, catalyzed by the enzyme phytoene synthase (Supplementary Table 2). Phytoene synthase gene (*crt*B), predicted in the genomics analysis, performs the condensation of two GGPP molecules, which are then converted into lycopene through desaturation and isomerization steps catalyzed by phytoene desaturase, zeta-carotene desaturase, and cis-carotene isomerase (Supplementary Table 2). Lycopene serves as a precursor for β-carotene (*m/z* 536.4392 [M]^+^, C_40_H_56_[M]^+^, Δ = 1.86 ppm) converted by the lycopene cyclase (CruA/P) (Supplementary Table 2). β-carotene is the starting point for the biosynthesis of other carotenoids like zeaxanthin (*m/z* 568.4246 [M]^+^, C_40_H_56_O_2_, Δ = −6.03 ppm), mediated by β-carotene hydroxylase (*crt*R) (Supplementary Table 2).

In *A. elenkinii* CCIBt3563, zeaxanthin is then converted into nostoxanthin (*m/z* 600.4154 [M]^+^, C_40_H_56_O_4_, Δ = −4.09 ppm). In these Pantanal strains, antheraxanthin (*m/z* 585.4231 [M + H]^+^, C_40_H_56_O_3_, Δ = 0.26 ppm), β-cryptoxanthin (*m/z* 553.4355 [M + H]^+^, C_40_H_56_O, Δ = 4.40 ppm), violaxanthin (*m/z* 601.4191 [M + H]^+^, C_40_H_56_O_4_, Δ = 2.10 ppm), and other compounds can be formed through the reversible activity of epoxidase and de-epoxidase enzymes in a process known as the violaxanthin cycle. β-cryptoxanthin is converted back into zeaxanthin by hydrogenase, which removes a hydrogen atom from the C3′ position of β-cryptoxanthin. β-carotene is also a precursor for the biosynthesis of echinenone and canthaxanthin, both produced by a β-carotene ketolase (*crt*S) and a hydroxylase (*Crt*R), which can convert echinenone to zeaxanthin (Supplementary Table 2). Echinenone can be hydroxylated to hydroxyechinenone by a hydroxylase enzyme. *A. elenkinii* CCIBt3563 produced β-carotene, canthaxanthin, and echinenone (Supplementary Table 4). Curiously, canthaxanthin was detected exclusively in this strain. Additionally, exclusive compounds detected by LC-MS/MS include myxoxanthophylls, myxol 2′-glucoside similar (*m/z* 746.4761, C_46_H_66_O_8_, Δ = 0.49 ppm), and β-apo-2′-carotenal (*m/z* 509.3717 [M + H]^+^, C_37_H_48_O, Δ = 2.32 ppm), which are directly related to enzymes involved in the xanthophyll cycle (Figure 3 and Supplementary Table 2).

Distinct carotenoid profiles, including glycosylated apocarotenoids, were identified in *A. pantanalense* CENA528, *Geminocystis* sp. CENA526, and *P. rosaneae* CENA516 (Figure 3D). Enzymes like lycopene cyclase (*cru*A/*cru*P) and carotenoid desaturases add complexity to the carotenoid structures, and carotenoid cleavage dioxygenases (*crt*S, CCD1, CCD4) form apocarotenoids (Supplementary Table 2). In *A. pantanalense* CENA528, there was a greater production of zeaxanthin, β-cryptoxanthin, and β-carotene (Supplementary Table 4). Putative compounds such as ketohydroxylycopene (*m/z* 566.4576 [M]^+^, C_40_H_54_O_2_), glycosyl-4,4′-diaponeurosporenoate (*m/z* 594.5125, C_36_H_50_O_7_) and 1,2-dihydrolycopene (*m/z* 538.4392 [M]^+^, C_40_H_58_) were also produced by this strain. CruA/CruP catalyzes lycopene cyclization, leading to 1,2-dihydrolycopene formation, while CCD1 and CCD4 cleave carotenoid molecules to form apocarotenoids. In *P. rosaneae* CENA516, β-carotene, zeaxanthin, and echinenone were the most abundant carotenes (Supplementary Table 4). Exclusively detected compounds include tetradehydro-2,2-diketo-beta-carotene (*m/z* 561.3655 [M + H]^+^, C_40_H_48_O_2_, Δ = 0.12 ppm) and an apocarotenoid (*m/z* 608.2908). However, *Geminocystis* sp. CENA526 was the only strain where β-carotene was not the most abundant carotenoid; instead, α-cryptoxanthin, zeaxanthin, and echinenone dominated. Several apocarotenoid glycosides (*m/z* 580.4880, *m/z* 585.4225, *m/z* 594.2759, *m/z* 620.5712, *m/z* 622.1915, *m/z* 624.5204, *m/z* 638.5671, *m/z* 664.4858, *m/z* 668.5379) and a putative phoenicoxanthin (*m/z* 581.4175 [M + H]^+^, C_40_H_52_O_3_) were detected exclusively in this strain (Figure 3 and Supplementary Table 4).

The strain *L. platensis* CENA597 is notable for also producing β-carotene, β-cryptoxanthin and antheraxanthin. The adonixanthin (*m/z* 582.4039 [M]^+^, C_40_H_54_O_3_, Δ = −3.61 ppm) and myxoxanthophyll compound (*m/z* 703.4564, C_44_H_63_O_7_, Δ = 0.60 ppm) were detected exclusively in this strain, likely related to lycopene cyclase (CruA/CruP) and β-carotenoid hydroxylase involved in xanthin myxol formation (Figure 3 and Supplementary Table 2). Another *L. platensis* strain isolated from Pantanal, CENA650 exclusively produced a xanthophyll compound (*m/z* 542.4881, C_40_H_62_, Δ = 5.43 ppm), and a carotene group compound (*m/z* 540.4738 [M]^+^, C_40_H_60_, Δ = −0.09 ppm) suggesting that lycopene cyclase (CruA/CruP) might be involved in carotenoid cyclization, forming tetrahydrolycopene. Additionally, lycopene derivatives are also present, likely as complex derivatives of the lycopene molecule. Both *Limnospira* strains CENA597 and CENA650, also produced in high abundance the compound annotated as 3,3′-ditetrahydropyranosyloxyisorenieratene (*m/z* 729.4725 [M + H]^+^, C_46_H_66_O_7_, Δ = −1.92 ppm), which is associated with the isorenieratene pigment biosynthesis pathway and the *crt*U gene (carotenoid phi/chi-ring synthase) predicted in both genomes (Supplementary Tables 2, 4).

## 4 Discussion

Terpenoids are generated from a complex process that involves several steps with enzymes in specific sequences of reactions and intermediates in the metabolic pathway (Liang et al., 2006). The genes and enzymes involved in the MEP pathway have already been found in several organisms and in cyanobacterial strains (Lichtenthaler, 2000; Pattanaik and Lindberg, 2015). This pathway is the main metabolic route for the biosynthesis of isoprenoids in prokaryotes. The genes related to the MEP pathway of terpene biosynthesis are considered conserved across cyanobacteria, with high similarity even between evolutionarily distant genera and simultaneously share homology with the mevalonate pathway in eukaryotes (Lange et al., 2000). As an essential metabolic route for cyanobacterial biology, inhibiting enzymes in this pathway causes lethal effects, mainly affecting photosynthesis and respiration and inhibiting oxidative activity (Pattanaik and Lindberg, 2015; Rudolf et al., 2021).

Carotenoid pigments play a crucial role in photosynthesis, capturing light and protecting against photo-induced damage (Hashimoto et al., 2018; Jacinavicius et al., 2019). In cyanobacteria, β-carotene is the primary carotenoid, along with zeaxanthin, canthaxanthin, β-cryptoxanthin, and echinenone (Takaichi, 2011). These strains share many enzymatic pathways related to terpenoid biosynthesis; however, the comparative analysis of terpenoid profiles among cyanobacterial strains performed in this study revealed significant biochemical diversity. Unique compounds were annotated in each extract from different Pantanal strains (Supplementary Table 2). The metabolomic analysis showed that the most crucial similarity in terpenoid production is not necessarily found among strains of the same species but was detected among strains from different families, such as *Geminocystis* sp. CENA526 and *L. platensis* CENA597, a unicellular and a filamentous cyanobacteria, respectively. The strains *Geminocystis* sp CENA526 and *L. platensis* CENA597, isolated from Salina Grande and Salina Centenário, respectively, showed a consistent presence of hydroxycarotenoids and other shared terpenoid classes (Figures 3C, D), which are metabolites commonly linked to photoprotective functions. At the same time, the results highlight metabolic variations within the same species, as seen by the higher number of different instead of equal terpenoid classes in CENA597 and CENA650, two strains of *Limnospira platensis* (Figure 3C). This observation demonstrates significant genetic and biochemical diversity in terpene production among cyanobacteria from tropical soda lakes, which appear to exhibit lineage-specific metabolomic adaptations that likely reflect genetic factors, niche specialization, and evolutionary responses to the extreme physicochemical parameters of their habitats. These adaptations can be observed in the *Synechococcus* PCC7002 and *Synechococcus elongatus* PCC7942, which present differences in sesquiterpene and monoterpene production due to variations in the terpene synthase enzymes (Chenebault et al., 2023). In addition, the differences in carotenoid composition relate to the presence or absence of specific genes and variations in the catalytic properties of key enzymes (Hirschberg and Chamovitz, 1994; Mochimaru et al., 2005; Takaichi and Mochimaru, 2007). Environmental conditions such as growth stage, light intensity, nitrogen source, and nitrogen concentration, as well as strain-specific characteristics within a species, also contribute to these phenomena (Olaizola and Duerr, 1990; Kłodawska et al., 2019; Llewellyn et al., 2020).

Terpenoid composition variation can be attributed to the presence or absence of specific carotenoid biosynthetic pathways and genes, as well as the distinct characteristics of the enzymes involved. As observed, all genomes share common genes for carotenoid production (*crt*B, *crt*P, *crt*Q, *crt*H, and *crt*R), and the presence of respective compounds appears conserved. This conservation might represent a core metabolome that depends on differential gene expression, regulatory mechanisms, or interactions with other genes, which are influenced by specific environmental conditions in Pantanal, such as salinity, solar radiation, or nutrient availability. Such correlations are observed when genes linked to specific pigment groups (*crt*S, *crt*W, *crt*X, and *crt*U) are distributed among different species (Supplementary Table 2), suggesting adaptive mechanisms, as they may relate to increased regulatory demands (e.g., light regulation) and other environmental characteristics (Liang et al., 2006; Schopf et al., 2013; Ma and Cui, 2022). Another condition that may influence the composition of different specific carotenoid compounds that these strains produce would be their responses to oxidative stress. Carotenoids can neutralize reactive oxygen species and scavenge free radicals (Montenegro et al., 2002). The carotenoid pigments found in this study stood out as more efficient in light capture under conditions of excess or scarcity of light (Stamatakis et al., 2014). *A. elenkinii* CCIBt3563, isolated from Salina da Reserva, exhibited a distinct xanthophyll profile not observed in the other strains. The accumulation of xanthophylls like zeaxanthin has been observed in the cytoplasmic membranes of *Synechococcus* cells grown under high irradiance conditions (Masamoto et al., 1999) and is likely associated with enhanced photoprotection and oxidative stress mitigation, crucial for survival under conditions of high irradiance. In contrast, *A. pantanalense* CENA528, from Salina Preta, displayed a comparatively lower diversity of terpenoids. This may suggest a narrower ecological amplitude or adaptation to different habitats, where a reduced repertoire of secondary metabolites is sufficient for persistence. Additionally, it might correlate with cyanobacterial blooms, given that each species adapts differently to light availability in the presence of dominant cyanobacteria, such as *A. elenkinii* and *L. platensis*, the reported main bloom-forming species in the Pantanal soda lakes (Andreote et al., 2018; Pellegrinetti et al., 2023).

The *crtB* gene encodes phytoene synthase, an essential enzyme in the carotenoid biosynthesis pathway. The enzyme phytoene synthase (CrtB) initiates the biosynthetic pathway of carotenoids, which is essential for producing various carotenoids. It catalyzes the head-to-head condensation of two molecules of geranylgeranyl pyrophosphate (GGPP), forming phytoene—the first committed step in carotenoid biosynthesis. Subsequent desaturation steps are catalyzed by enzymes such as phytoene desaturase (*crt*I, or its cyanobacterial equivalents *crtP* and *crtQ*), which convert phytoene into carotenoids like neurosporene or lycopene (Sandmann, 2002; Kato et al., 2016). Phytoene has increasingly been associated with photoprotective activities. While phytoene has an absorbance maximum of around 286 nm, effectively protecting cells from UV-B radiation, phytofluene absorbs at 348 nm in the UV-A region (Meléndez-Martínez et al., 2019). Combining these carotenoids in a bioproduct might represent an eco-friendly option for chemical sunscreens related to commercial options using mycosporine-like amino acids for photoprotection (Sen and Mallick, 2021). The phytoene synthase gene found in the Pantanal strains showed evolutionary similarity with strains from brackish water on the Baltic Sea, hypersaline lakes in Tanzania, and freshwater lakes in Brazil and China. These diverse environmental conditions might pose different abiotic stresses, but UV exposure is obligatory for photosynthetic organisms. Recent data show that both *L. platensis* CENA597 and CENA650, as well as *A. elenkinii* CCIBt3563, lack the genetic arrangement for mycosporine-like amino acid production, failing to biosynthesize these compounds even when exposed to UV radiation (Dextro et al., 2023). Therefore, it is possible to assume that other photoprotective strategies, such as using carotenoids, are employed by these strains to survive and even form blooms in these highly UV-exposed habitats.

Strains *L. platensis* CENA597, *L. platensis* CENA650, and *A. elenkinii* CCIBt3563 likely share the same adaptive mechanisms that grant endurance in habitats highly exposed to UV radiation, which affects the photosynthetic efficiency of cyanobacteria (Jacinavicius et al., 2021). The xanthophyll cycle was highly active in these strains, with the identification of unique compounds present in this pathway (xanthophyll, adonixanthin, myxoxanthophyll, canthaxanthin, and 2′-fucosyl-4-hydroxy-myxol-2′-fucoside). Xanthophylls are oxygen-containing carotenoids that include functional groups such as aldehydes, carboxyl, and epoxides (Maoka, 2020). Xanthophylls have potent antioxidant activity that contributes to the integrity of the thylakoid membrane against excess light, which makes these molecules significant photoprotective compounds (Srivastava et al., 2022). Exposure to UV-B led to changes in carotenoid transcription regulation, affecting carotenoid synthesis, photoprotection, and cleavage, with increased expression of ketolase-related genes resulting in higher concentrations of echinenone, canthaxanthin, and myxoxanthophyll that reduced DNA damage and oxidative stress and absorbed radiation, potentially promoting DNA repair (Ehling-Schulz et al., 1997; Llewellyn et al., 2020). The pigments β-cryptoxanthin, echinenone, canthaxanthin, astaxanthin, and zeaxanthin have antioxidant properties, acting against reactive oxygen species within the cell, protecting molecules from oxidative stress (Gruszecki and Strzałka, 2005; Sedoud et al., 2014; Pereira et al., 2021). Additionally, xanthophylls have anti-inflammatory and antitumor activities due to their antioxidant properties and double bonds in their chemical structure, highlighting their commercial appeal for biotechnology in the cosmetics and food industries (Álvarez et al., 2014; Pereira et al., 2021).

Genes involved in the carotenogenesis pathway, from geranylgeranyl-diphosphate to lycopene, are also upregulated following exposure to UV-B light radiation (Llewellyn et al., 2020). Lycopene acts as a precursor to cyclic β-carotenes, as well as to xanthophyll carotenoids, with lycopene cyclase being responsible for catalyzing the formation of cyclic end groups of these carotenoids (Domonkos et al., 2013). Various types of lycopene cyclase, including the first identified type, *crt*L (*crt*L-b, LCY-b), have been confirmed in cyanobacteria (Sugiyama and Takaichi, 2020). Cyanobacteria whose genome does not contain genes for lycopene beta cyclase (*crt*L) have a gene like *cru*A (ortholog), which may perform a similar or compensatory function to lycopene cyclase (Maresca et al., 2007). *cru*A/c*ru*P was detected in all strains of the Pantanal genome. The homologous genes of *cru*A and *cru*P are widely distributed in some cyanobacterial species’ genomes; however, few reports demonstrate lycopene cyclase activity in products (Sugiyama and Takaichi, 2020). Modifications to carotenes can produce glycosylated carotenoids, which are also very common in cyanobacteria (Hirschberg and Chamovitz, 1994; Takaichi et al., 2005). Specific glycosylated carotenoids, likely as complex derivatives of the lycopene molecule, are present in *Geminocystis* sp. CENA526 and *A. pantanalense* CENA528. Cyanobacteria synthesize these apocarotenoids through a complex biosynthetic pathway involving carotenogenic enzymes via the activity of carotenoid cleavage dioxygenases (CCDs) (Liang et al., 2017; Hambly et al., 2021). These activities reflect the complexity and diversity in carotenoid biosynthesis among these strains. In cyanobacteria, apocarotenoids play crucial roles in protecting photosynthetic machinery from oxidative damage caused by excessive light, capturing light, and transferring energy to the photosynthetic complexes; still, many apocarotenoids have biological functions that remain challenging to determine and are not yet fully understood (Liang et al., 2017).

The hopanoid bacteriohopanetetrol was exclusively annotated in the *Geminocystis* sp. CENA526 strain, suggesting a variation in lipid composition and osmotolerance between the strains (Supplementary Table 2). Hopene and other hopanoids occur in a wide range of Gram-positive and Gram-negative bacteria, supporting small bacterial adaptation to extreme conditions, like high temperatures and pH gradients (Carvalho and Fernandes, 2010). The presence of detectable hopanoids likely protects the membrane in *Geminocystis* sp. CENA26, suggesting an adaptive strategy to the Pantanal lakes, where high salinity and intense UV radiation occur. *sqh*C is a primitive condition gene found in several modern bacterial genera, but it is estimated to occur in only 5–10% of all bacterial species (Kharbush et al., 2013). The synteny analysis for the predicted SHC enzyme gene showed an identity score of around 60% between the Pantanal strains and the sequences of other cyanobacteria. This analysis corroborates with the *sqh*C tree (Supplementary Figure 5) and the works of Pearson et al. (2007) and Kharbush et al. (2013), where SHCs have a similarity between 45 and 55% within cyanobacterial groups, with an average of 60% similarity between the closest taxonomical subgroups and about 90% for species within the same genus. A study with environmental and culturable samples suggests that possessing a squalene hopene cyclase (SHC) protein implies that cyanobacterial strains in culturable conditions will have detectable levels of bacteriohopanepolyols (BHPs) (Talbot et al., 2008, 2016). Additionally, structural variants of 2-methyl-BHPs, similar to those found for *Geminocystis* sp. CENA526 is present in different cyanobacterial mats from hypersaline marine lagoons in Australia and Mexico (Talbot et al., 2008). This difference reflects the structural and genetic diversity caused by environmental gradients that these organisms face. The predicted clusters showed similarity only for the SHC enzyme since the evaluated genera are not phylogenetically close to each other, thus indicating that this gene might be conserved among the entire phylum Cyanobacteria. In the phylogenetic analysis of *sqh*C, taxonomical similarity was observed, especially in grouping the gene copies from the *Geminocystis* genus, with sequence similarity of over 80%, forming a branch with common ancestry (Supplementary Figure 5). For the homologs genes annotated in *A. pantanalense* CENA528 and *P. rosaneae* CENA516, which does not present detectable production of hopanoids, that grouped with strains isolated from hot springs and freshwater samples, it can be argued that a better evolutionary resolution might only be achieved with further description of cyanobacterial diversity from wide environmental backgrounds, improving possible inferences between shared gene history in cyanobacteria (Dextro et al., 2021).

In addition to tetraterpenoids (carotenoids) and triterpenoids (hopanoids), meroterpenoids (ubiquinones) were also detected. The evolutionary trajectory of ubiquinone pathways suggests that the first set of ubiquinone biosynthetic enzymes were present in cyanobacteria that produced phylloquinone, so the current genes of these metabolic pathways found in other prokaryotes and eukaryotes are homologs inherited from cyanobacteria (Degli Esposti, 2017). Tocopherol was detected in all strains and has antioxidant activity, protecting membranes against the auto-oxidation of polyunsaturated fatty acids (Inoue et al., 2011). Meanwhile, menaquinone and phylloquinone (vitamin K) participate in photosystem I electron transfer (Gross et al., 2006; Sadre et al., 2012). These substances produced by cyanobacteria have the potential to be incorporated into nutraceuticals and pharmaceuticals, fortifying food for human or animal supplementation and acting as natural antioxidants (Kawamukai, 2018).

As observed, the intensity and quality of solar radiation might influence the production of terpenoids, which play a crucial role in protection against UV damage and oxidation. Likewise, salinity and nutrient changes cause carotenoid composition variations (Paliwal et al., 2015). Extreme conditions, such as those found in the Pantanal biome, with high salt concentrations, pH variations, or low water availability, can stimulate the production of specific carotenoids that help organisms survive in these adverse conditions. This biochemical diversity is the main factor in Cyanobacteria’s success in colonizing distinct habitats. Also, these carbon pathways play a crucial role in the tropical soda lakes as cyanobacterial blooms mediate carbon and energy fluxes, where Cyanobacteria represent a leading agent in supporting the heterotrophic bacterial community, particularly during the dry season (Cotta et al., 2022).

## 5 Conclusion

The genomes of cyanobacterial strains isolated from the soda lakes of the Pantanal biome were mined for genes associated with terpene and terpenoid biosynthesis, identifying several homologs. The studied strains shared many enzymatic pathways related to terpenoid biosynthesis. Still, they mainly differed in their responses to the production of tetraterpenoids, results corroborated by the chemical profile of the samples. Synteny and phylogenetic analysis of critical enzymes such as scalene hopene cyclase and phytoene synthase highlighted sequence conservation and shared evolutionary histories with cyanobacteria from diverse environments. Metabolomic analysis revealed significant diversity and specificity in carotenoid profiles, with unique features contributing to the strains’ adaptation to extreme conditions in the soda lakes. This study links genome mining with metabolomics, which serves as a starting point for research focused on biotechnological endpoints. The detection of bioactive compounds, including carotenoids, hopanoids, and tocopherols, further highlights the biotechnological potential of these strains. This potential extends to applications in pharmaceuticals, nutraceuticals, and cosmetics, opening new avenues for drug development and health supplements. The unique metabolic adaptations and biosynthetic pathways identified in these cyanobacteria provide a valuable foundation for further exploring their metabolites’ ecological roles and bioprospecting potential, especially under extreme environmental conditions.

## Data Availability

The genomic sequences for this study have been deposited and are available in the NCBI database under the following accession numbers: *Alkalinema pantanalense* CENA528 (JBLZFX000000000), *Anabaenopsis elenkinii* CCIBt3563 (CP063311), *Geminocystis* sp. CENA526 (JBLZFY000000000), *Limnospira platensis* CENA597 (CP185278), *Limnospira platensis* CENA650 (JBLZFW000000000), and *Pantanalinema rosanae* CENA516 (JBLZFZ000000000).

## Footnotes

1
http://gnps.ucsd.edu

2
http://www.chemcalc.org

3
http://carotenoiddb.jp/index.html
