# Supplementary material for: Genetic and biochemical diversity of terpene biosynthesis in cyanobacterial strains from tropical soda lakes
Source: Front Microbiol. 2025 Jul 4;16:1582103. doi: 10.3389/fmicb.2025.1582103 (PMC12271159; doi:10.3389/fmicb.2025.1582103)
Supplement: Supplementary file 1 [file Supplementary_file_1.zip › Supplementary Figures.PDF]

Supplementary Material

Genetic and biochemical diversity of terpene biosynthesis in cyanobacterial strains from tropical soda lakes

Mauricio J. Machado[1]; Fernanda R. Jacinavicius[1]; Rhuana V. Médice[2]; Rafael B. Dextro[1]; Anderson M. T. Feitosa[1]; Marcio B. Weiss[2]; Thierry A. Pellegrinetti[1,3]; Simone R. Cotta[4]; Camila M. Crnkovic[2]; Marli F. Fiore[1]**

[1] Center for Nuclear Energy in Agriculture, University of São Paulo, Piracicaba, SP, Brazil

[2] School of Pharmaceutical Sciences, University of São Paulo, São Paulo, SP, Brazil

[3] Faculty of Agricultural Sciences and Food, Université Laval, Québec City, QB, Canada

[4] "Luiz de Queiroz" College of Agriculture, University of São Paulo, Piracicaba, SP, Brazil

*** Correspondence:**
Marli F. Fiore
fiore@cena.usp.br

1 Supplementary Figures

Figure S1. MEP biosynthetic pathway genes present in genomes manually annotated and predicted by the BlastKOALA tool through biosynthetic pathway recovery. The dash crossed by two lines represents discontinuity or long chains between genes

Figure S2. Venn diagram shows the intersection of the number of proteins orthologous between the genomes of cyanobacterial strains isolated from Pantanal, Brazil.

Figure S3. Synteny analysis for the scalene hopene cyclase gene, for the gene clusters predicted by the AntiSMASH platform most similar to the study genomes.

Figure S4. Maximum-likelihood phylogenetic tree of the phytoene synthase gene (crtB) found in *Anabaenopsis elenkinii* CCIBt3563, *Limnospira platensis* CENA597 and *Limnospira platensis* CENA650 to other strains of cyanobacteria from NCBI that presented crtB homologs, with accession number in parentheses.

Figure S5. Maximum-likelihood phylogenetic tree of the scalene hopene cyclase genes (sqhC) found in *Alkalinema pantanalense* CENA528, *Pantanalinema rosanae* CENA516 and *Geminocystis* sp. CENA526 to other strains of cyanobacteria from NCBI that presented sqhC homologs, with accession numbers in parentheses.

Figures S6 to S16. Ion product mass spectra of protonated terpenoids from cyanobacterial strains isolated from the Pantanal, Brazil. Arrows indicate the presence of characteristic ions within the molecules identified. Nodes indicate the strains in which the compounds were detected.

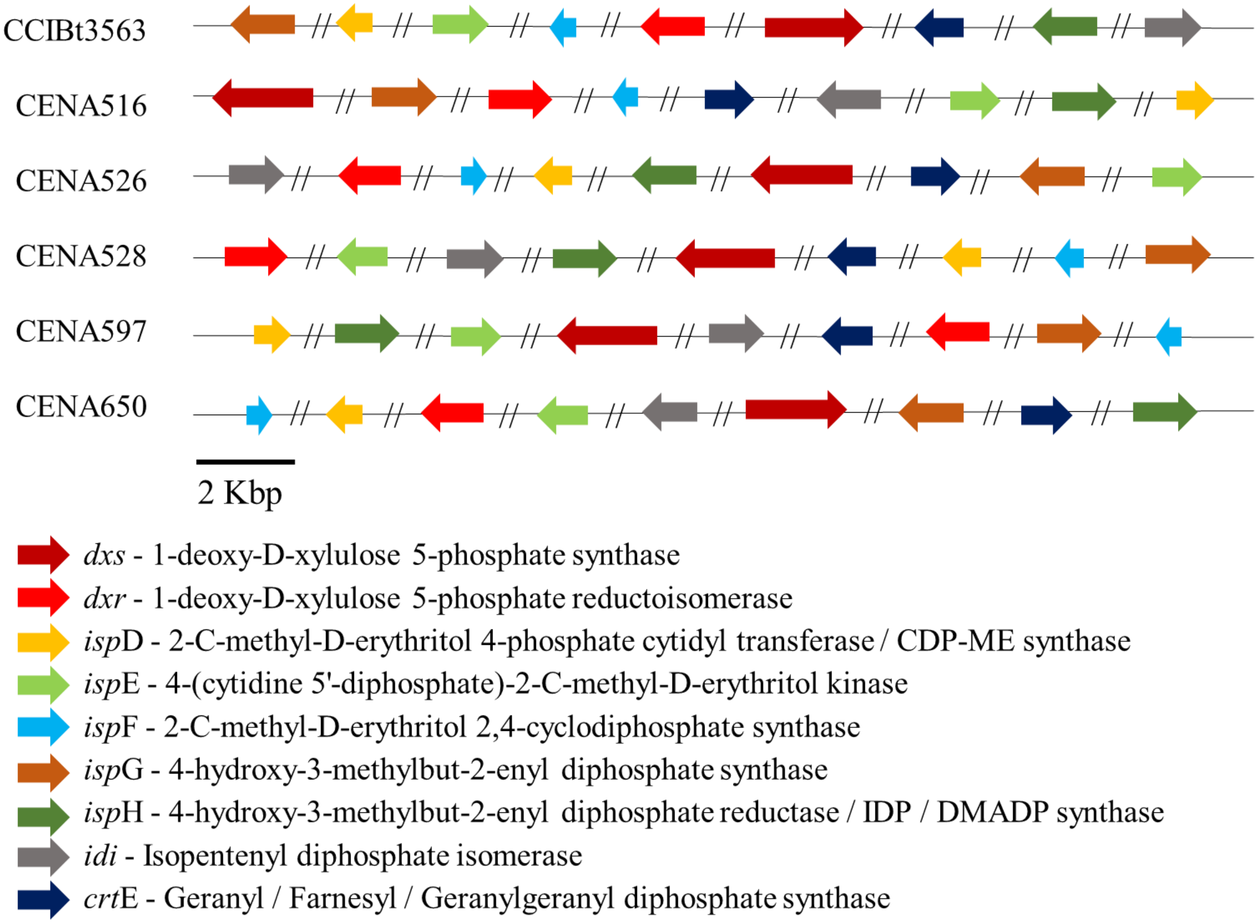

Figure S1. MEP biosynthetic pathway genes present in genomes manually annotated and predicted by the BlastKOALA tool through biosynthetic pathway recovery. The dash crossed by two lines represents discontinuity or long chains between genes

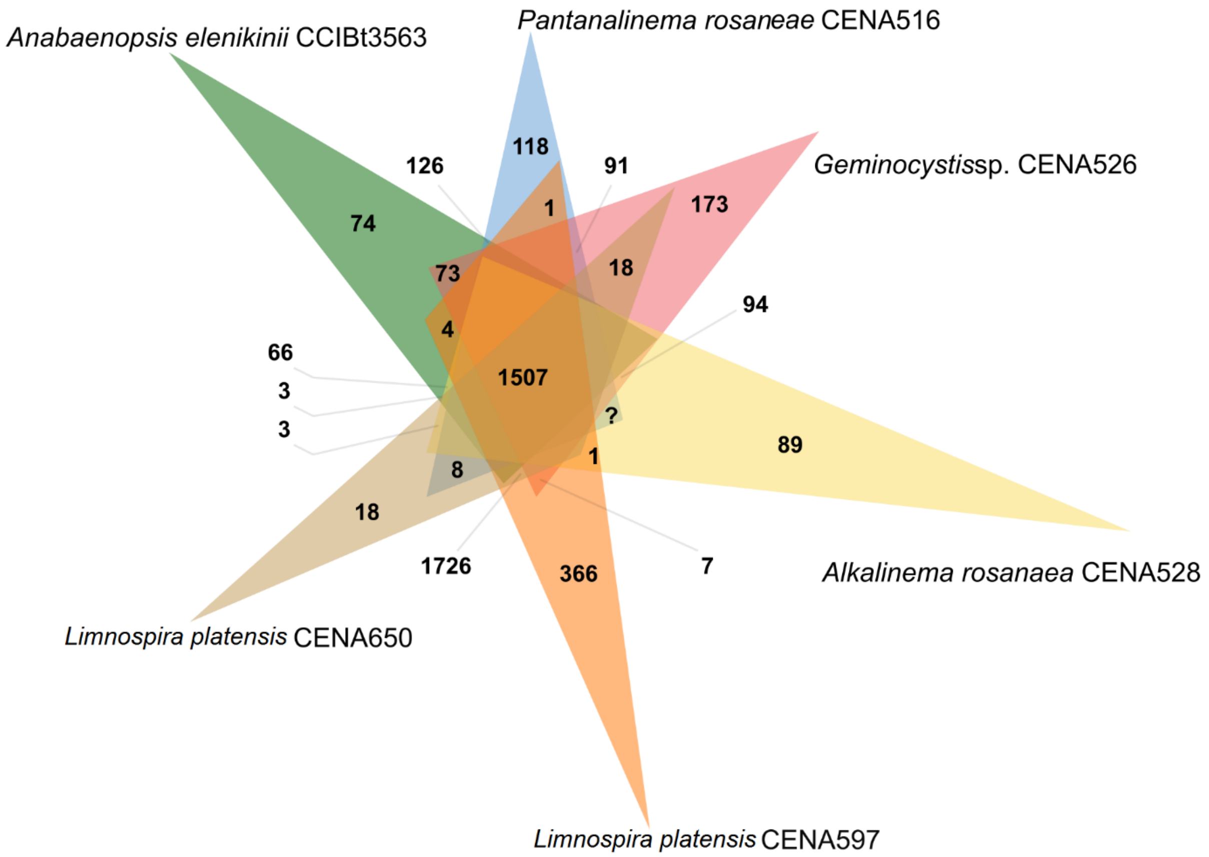

Figure S2. Venn diagram shows the intersection of the number of proteins orthologous between the genomes of cyanobacterial strains isolated from Pantanal, Brazil.

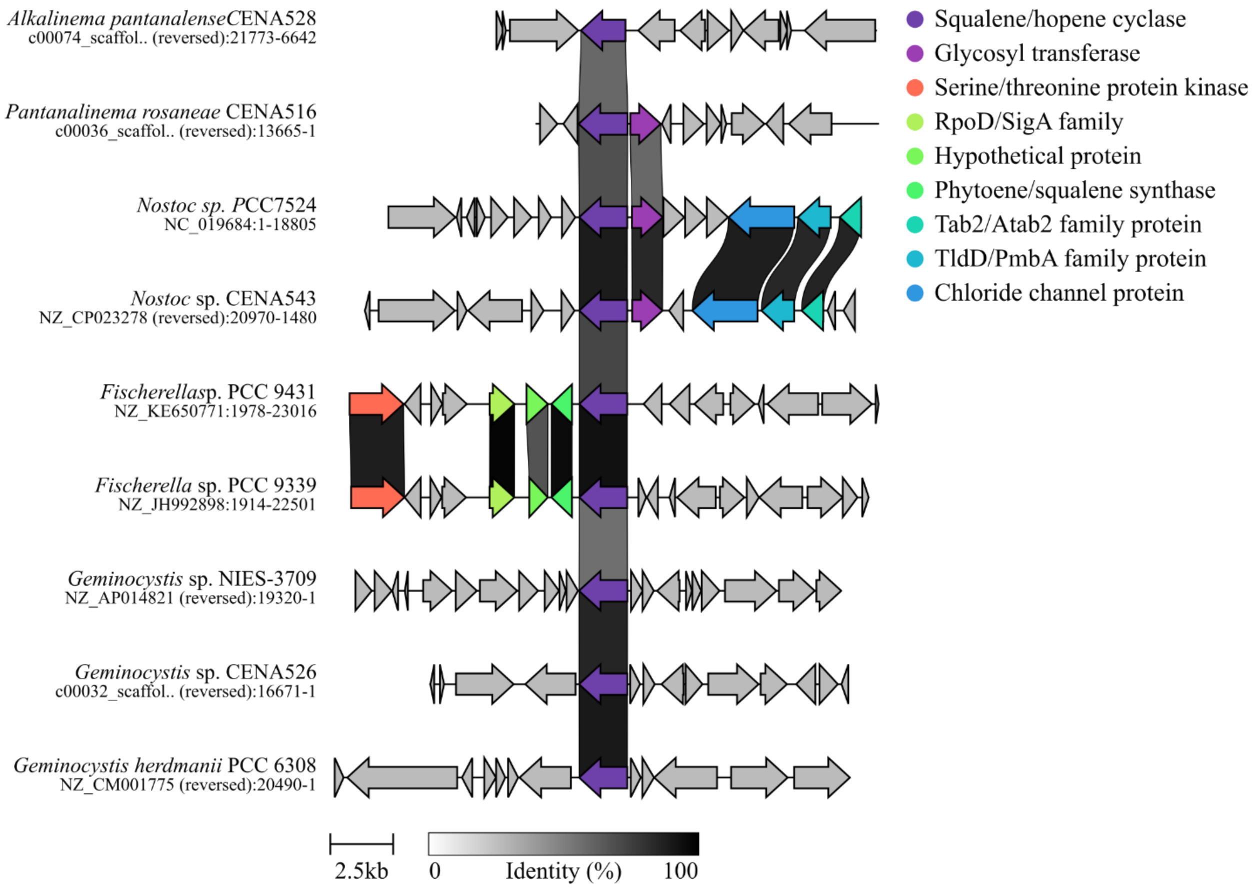

Figure S3. Synteny analysis for the scalene hopene cyclase gene, for the gene clusters predicted by the AntiSMASH platform most similar to the study genomes.

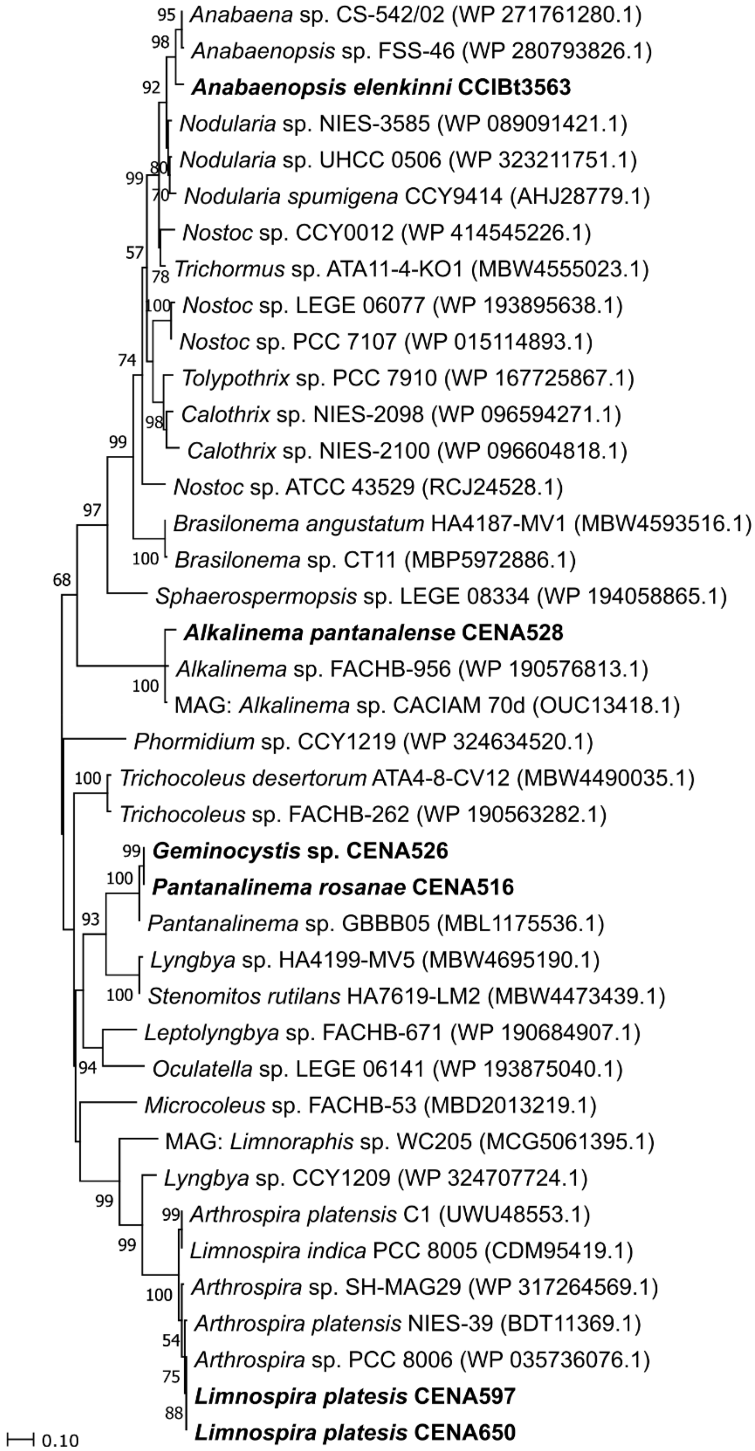

Figure S4. Maximum-likelihood phylogenetic tree of the phytoene synthase gene (*crt*B) found in *Alkalinema pantanalense* CENA528 (JBLZFX000000000), *Anabaenopsis elenkinii* CCIBt3563 (CP063311), *Geminocystis* sp. CENA526 (JBLZFY000000000), *Limnospira platensis* CENA597 (CP185278), *Limnospira platensis* CENA650 (JBLZFW000000000), and *Pantanalinema rosanae* CENA516 (JBLZFZ000000000) in relation to other strains of cyanobacteria from NCBI that presented *crt*B homologs, with accession number in parentheses. This analysis involved 40 amino acid sequences and a bootstrap of 1000 replicates.

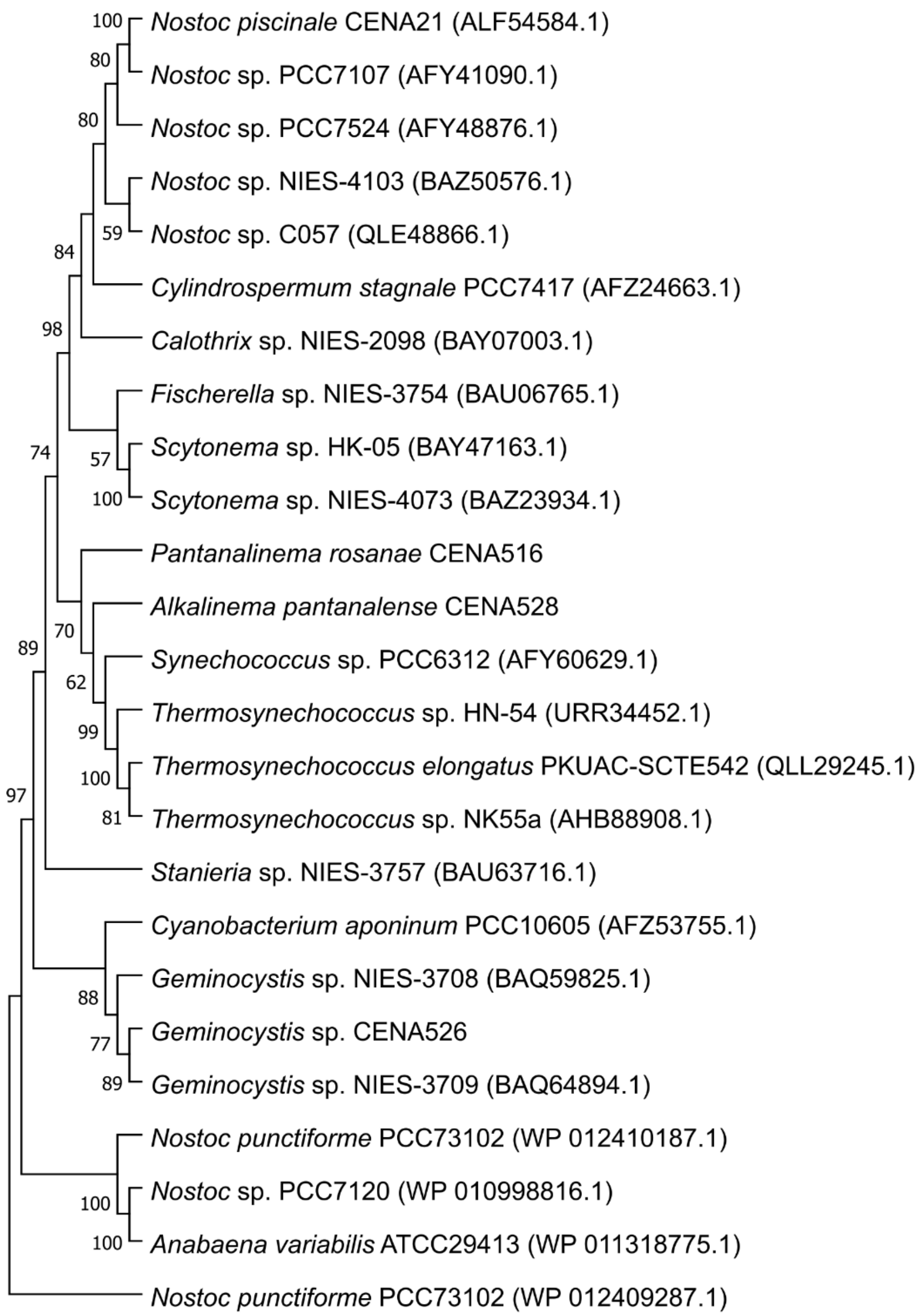

Figure S5. Maximum-likelihood phylogenetic tree of the scalene hopene cyclase genes (*sqh*C) found in *Alkalinema pantanalense* CENA528 (JBLZFX000000000), *Geminocystis* sp. CENA526 (JBLZFY000000000) , and *Pantanalinema rosanae* CENA516 (JBLZFZ000000000) in relation to other strains of cyanobacteria from NCBI that presented *sqh*C homologs, with accession number in parentheses. This analysis involved 25 amino acid sequences and a bootstrap of 1000 replicates.

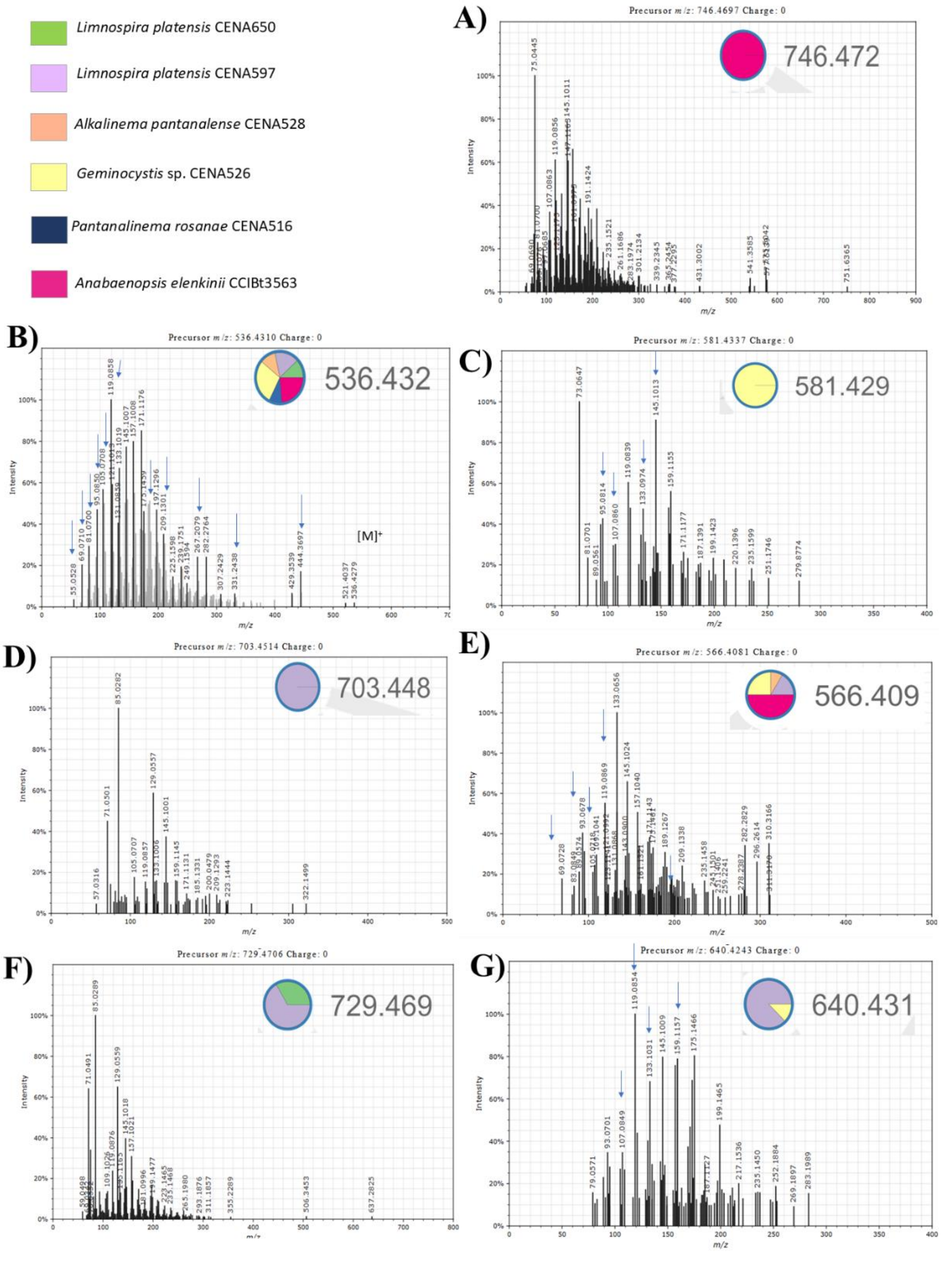

Figure S6. Ion product mass spectra of protonated terpenoids from cyanobacterial strains isolated from the Pantanal, Brazil. **A)** 746.472; **B)** 536.432; **C)** 581.429; **D)** 703.448; **E)** 566.409; **F)** 729.469; **G)** 640.43. Arrows indicate the presence of characteristic ions within the molecules identified. Nodes indicate the strains in which the compounds were detected.

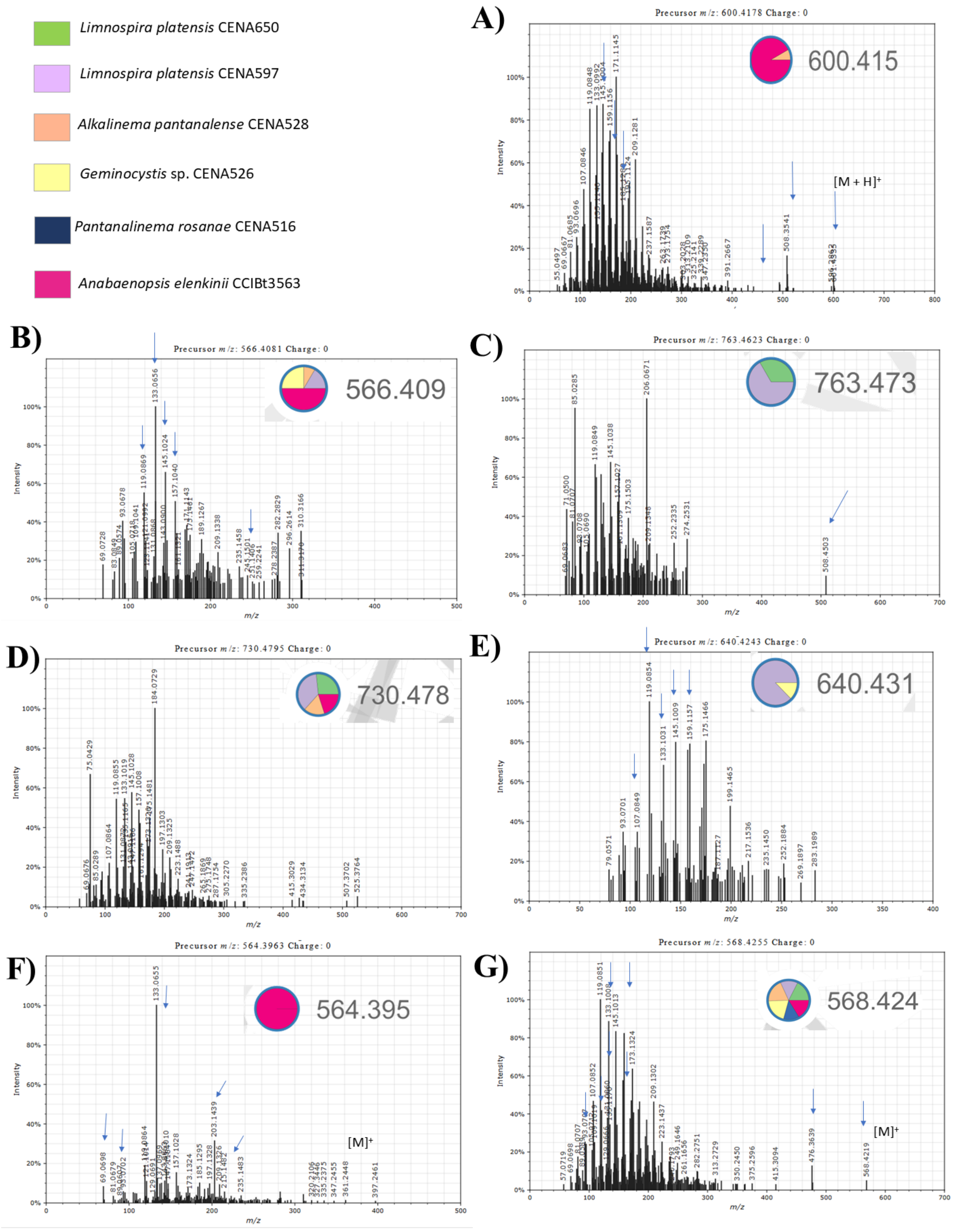

Figure S7. Ion product mass spectra of protonated terpenoids from cyanobacterial strains isolated from the Pantanal, Brazil. **A)** 600.415; **B)** 566.409; **C)** 763.473; **D)** 730.478; **E)** 640.431; **F)** 566.395; **G)** 568.424. Arrows indicate the presence of characteristic ions within the molecules identified. Nodes indicate the strains in which the compounds were detected.

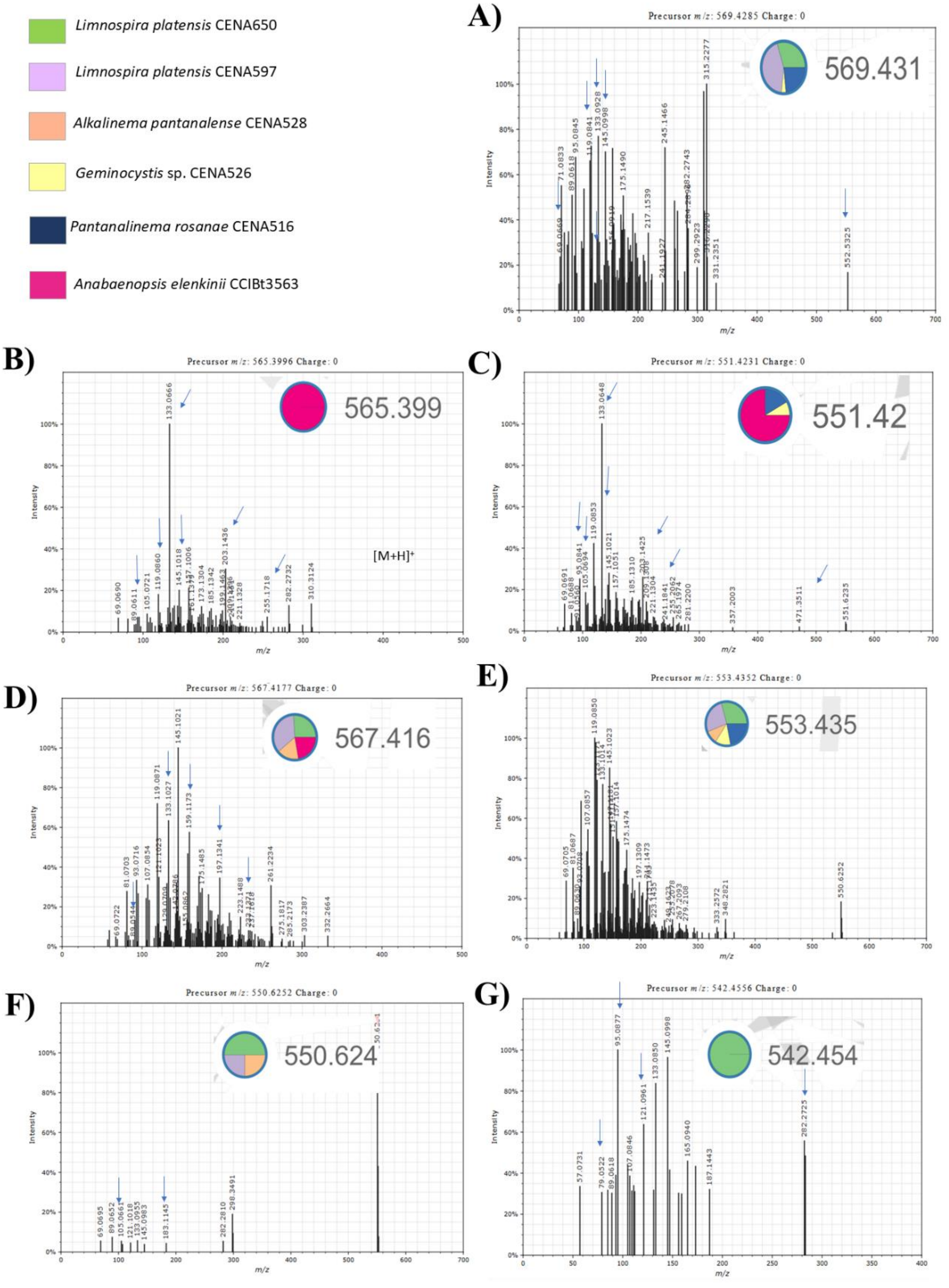

Figure S8. Ion product mass spectra of protonated terpenoids from cyanobacterial strains isolated from the Pantanal, Brazil. **A)** 569.431; **B)** 565.399; **C)** 551.420; **D)** 567.416; **E)** 553.435; **F)** 550.624; **G)** 542.454. Arrows indicate the presence of characteristic ions within the molecules identified. Nodes indicate the strains in which the compounds were detected.

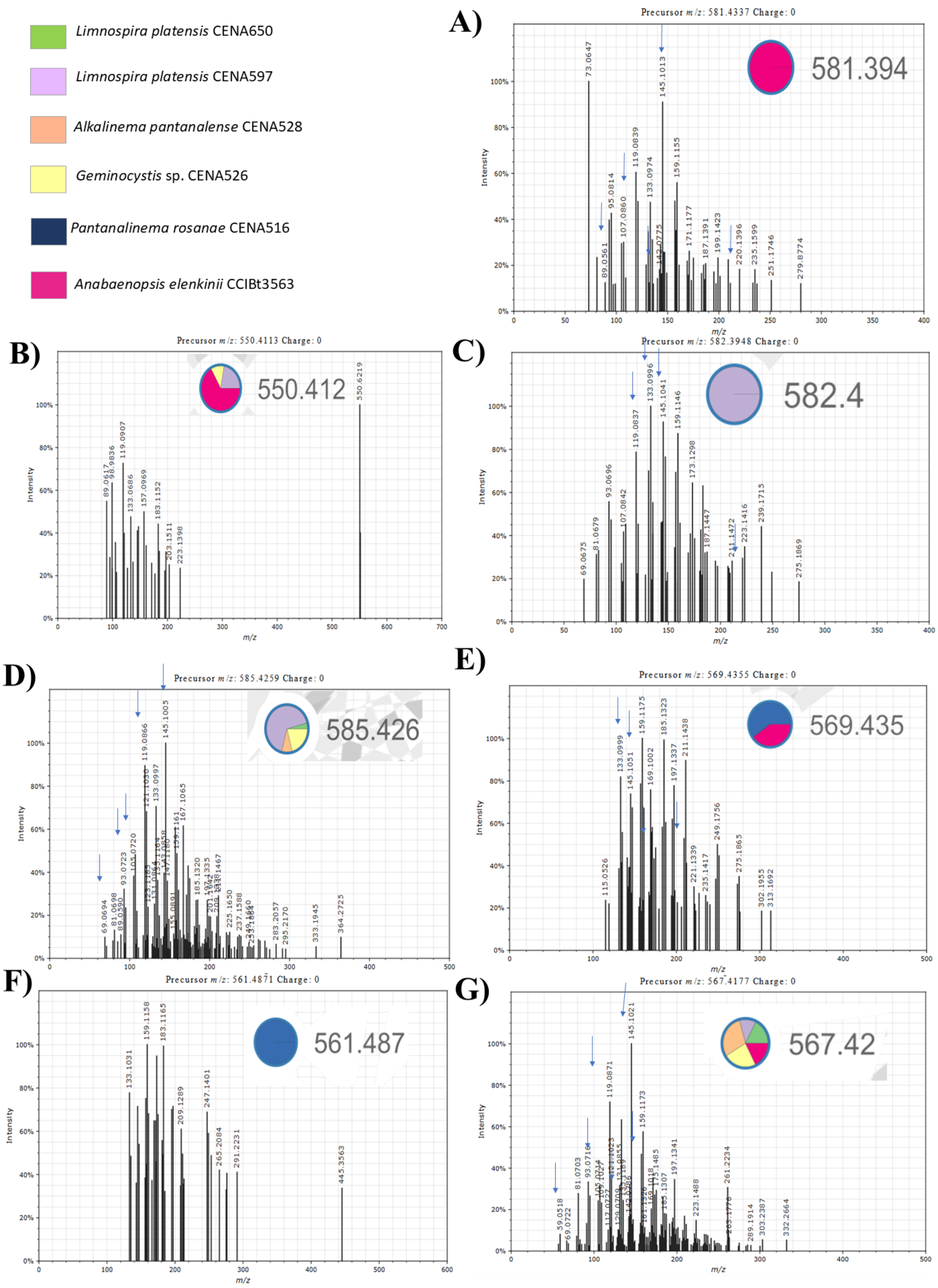

Figure S9. Ion product mass spectra of protonated terpenoids from cyanobacterial strains isolated from the Pantanal, Brazil. **A)** 581.394; **B)** 550.412; **C)** 582.4; **D)** 585.426; **E)** 569.435; **F)** 561.487; **G)** 567.42. Arrows indicate the presence of characteristic ions within the molecules identified. Nodes indicate the strains in which the compounds were detected.

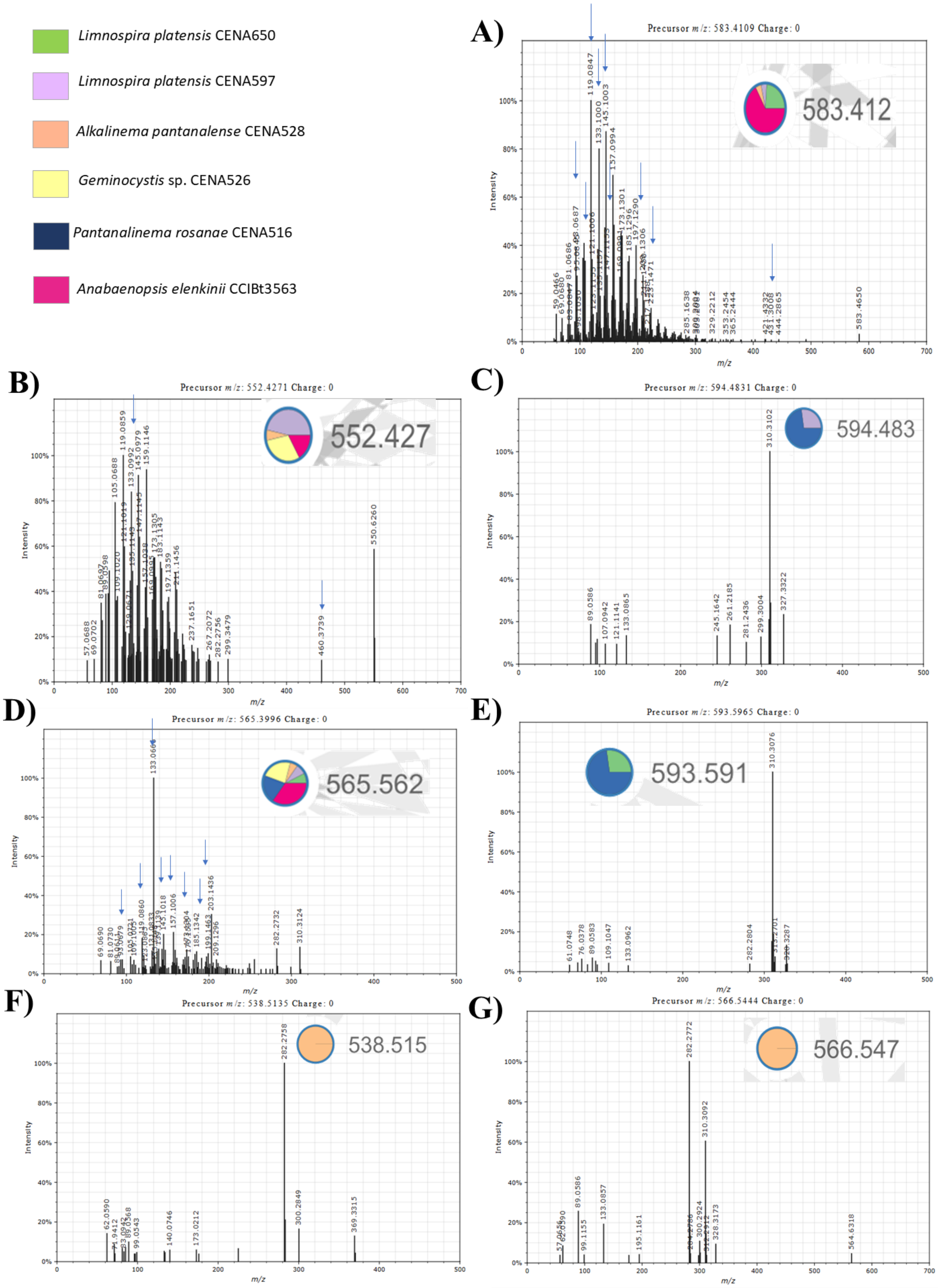

Figure S10. Ion product mass spectra of protonated terpenoids from cyanobacterial strains isolated from the Pantanal, Brazil. **A)** 583.41; **B)** 552.427; **C)** 594.483; **D)** 565.562; **E)** 593.591; **F)** 538.515; **G)** 566.547. Arrows indicate the presence of characteristic ions within the molecules identified. Nodes indicate the strains in which the compounds were detected.

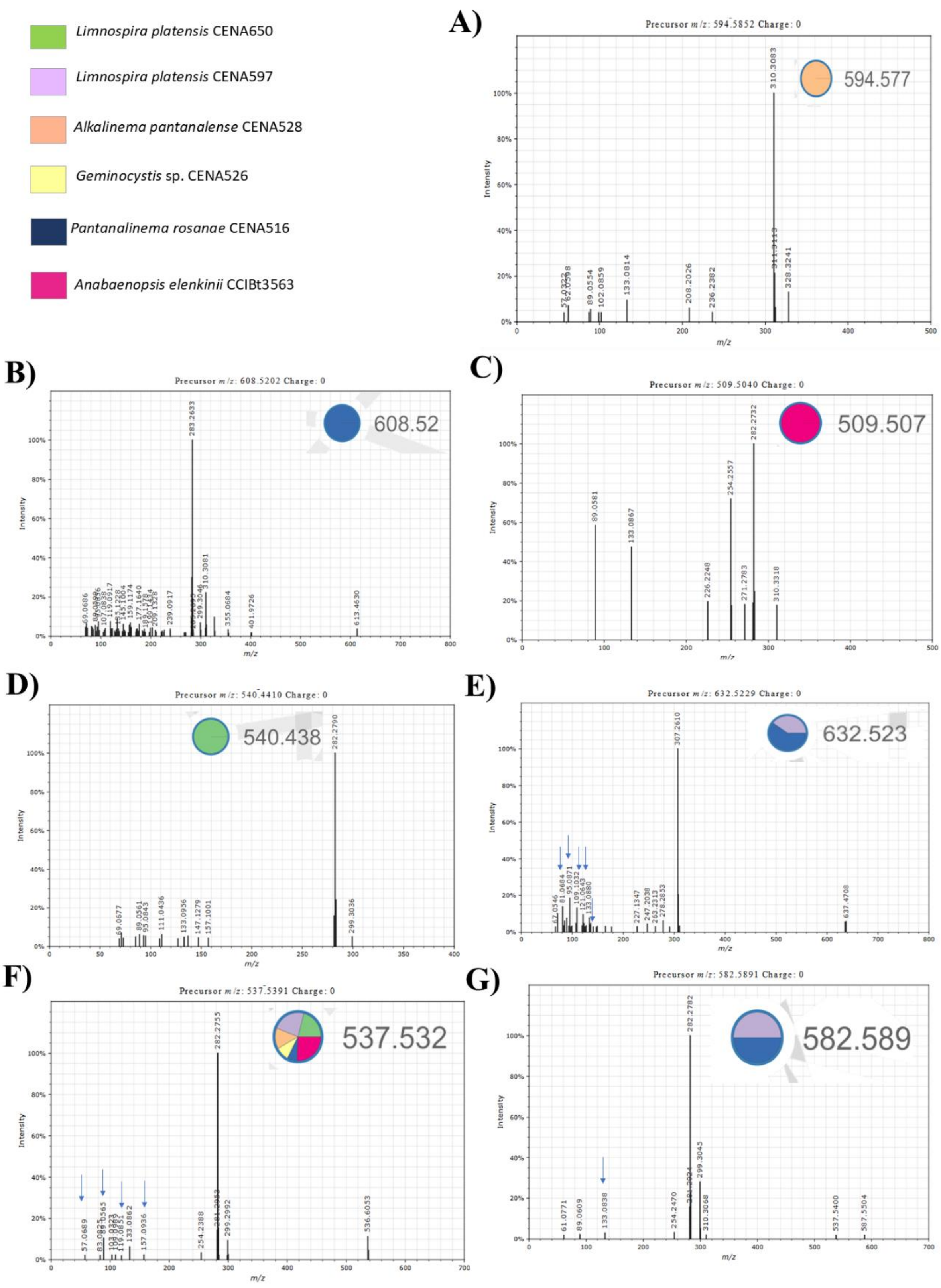

Figure S11. Ion product mass spectra of protonated terpenoids from cyanobacterial strains isolated from the Pantanal, Brazil. **A)** 594.577; **B)** 608.52; **C)** 509.507; **D)** 540.438; **E)** 632.523; **F)** 537.532; **G)** 582.583. Arrows indicate the presence of characteristic ions within the molecules identified. Nodes indicate the strains in which the compounds were detected.

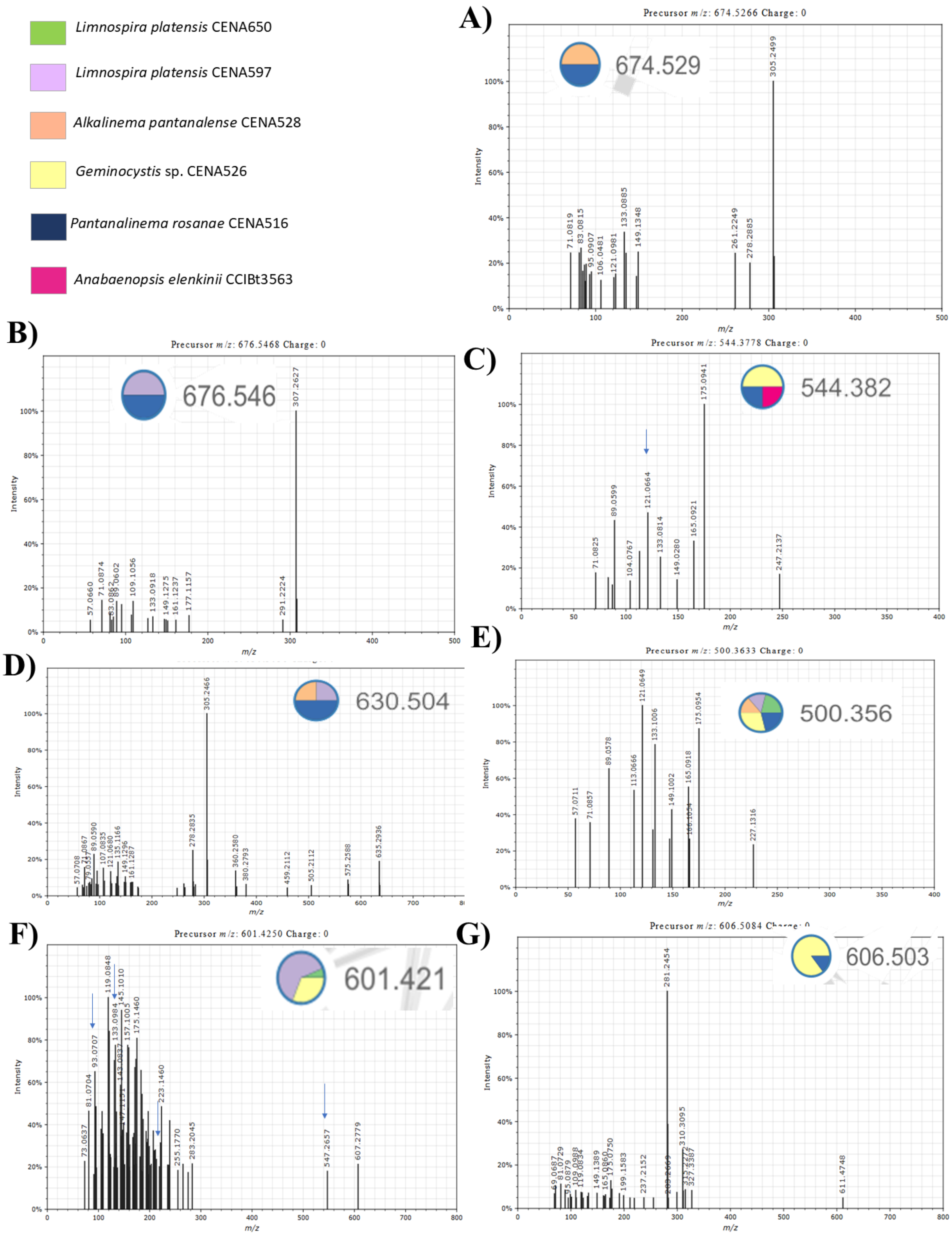

Figure S12. Ion product mass spectra of protonated terpenoids from cyanobacterial strains isolated from the Pantanal, Brazil. **A)** 674.529; **B)** 676.546; **C)** 544.382; **D)** 630.504; **E)** 500.356; **F)** 601.421; **G)** 606.503. Arrows indicate the presence of characteristic ions within the molecules identified. Nodes indicate the strains in which the compounds were detected.

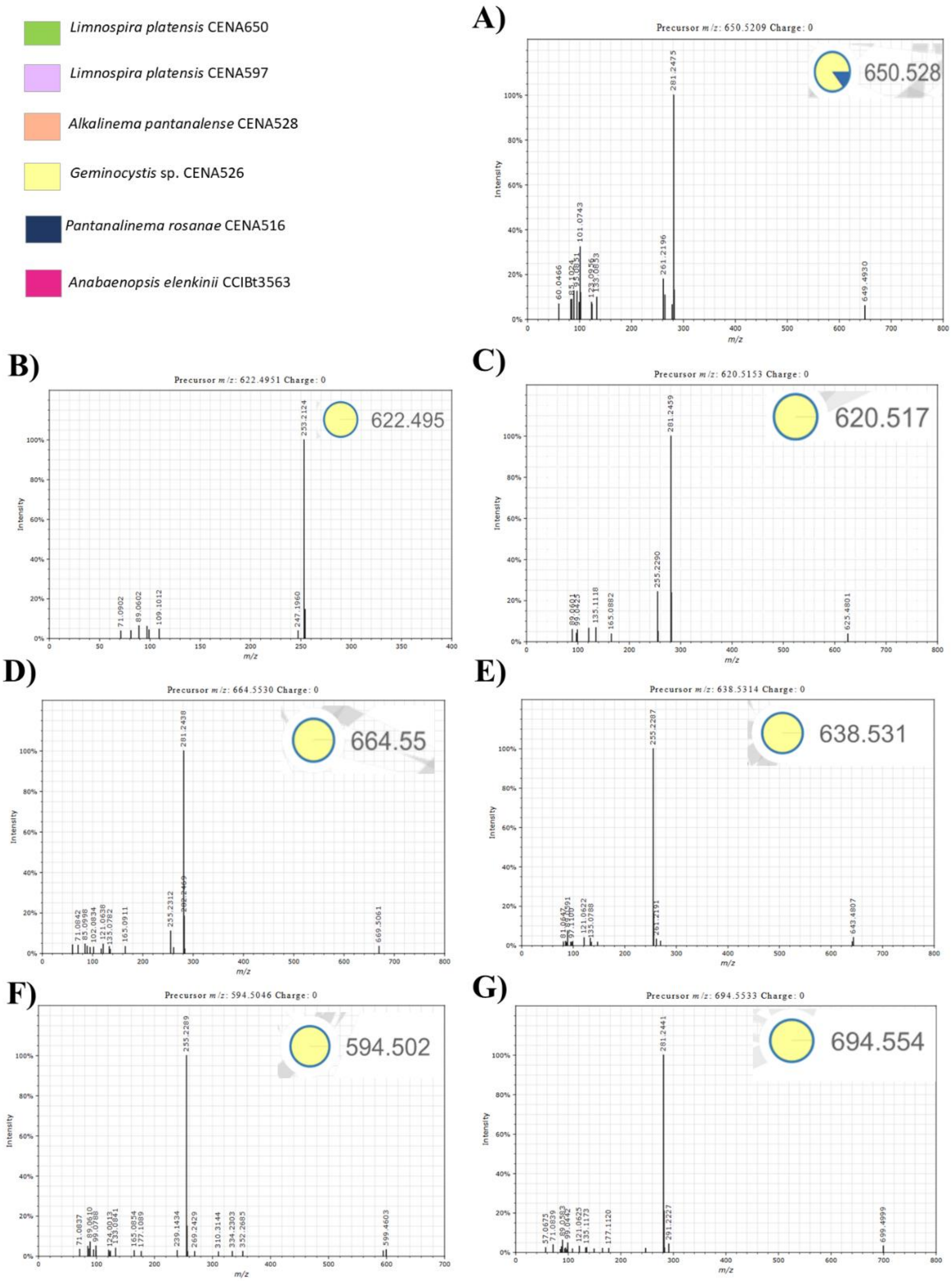

Figure S13. Ion product mass spectra of protonated terpenoids from cyanobacterial strains isolated from the Pantanal, Brazil. **A)** 650.528; **B)** 622.495; **C)** 620.517; **D)** 664.55; **E)** 638.531; **F)** 594.502; **G)** 694.554. Arrows indicate the presence of characteristic ions within the molecules identified. Nodes indicate the strains in which the compounds were detected.

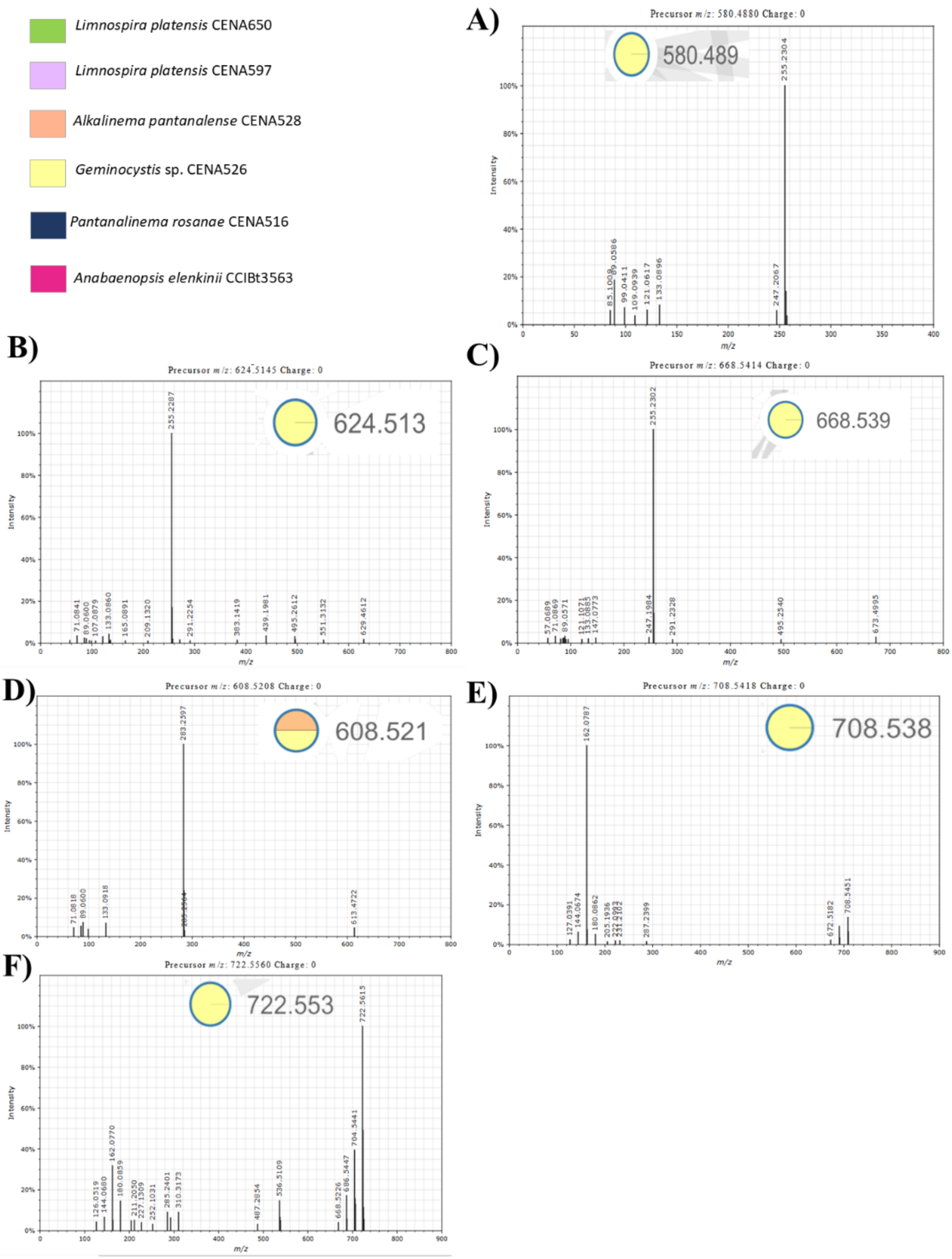

Figure S14. Ion product mass spectra of protonated terpenoids from cyanobacterial strains isolated from the Pantanal, Brazil. **A)** 580.489; **B)** 624.513; **C)** 668.539; **D)** 608.521; **E)** 708.538; **F)** 722.553. Arrows indicate the presence of characteristic ions within the molecules identified. Nodes indicate the strains in which the compounds were detected.

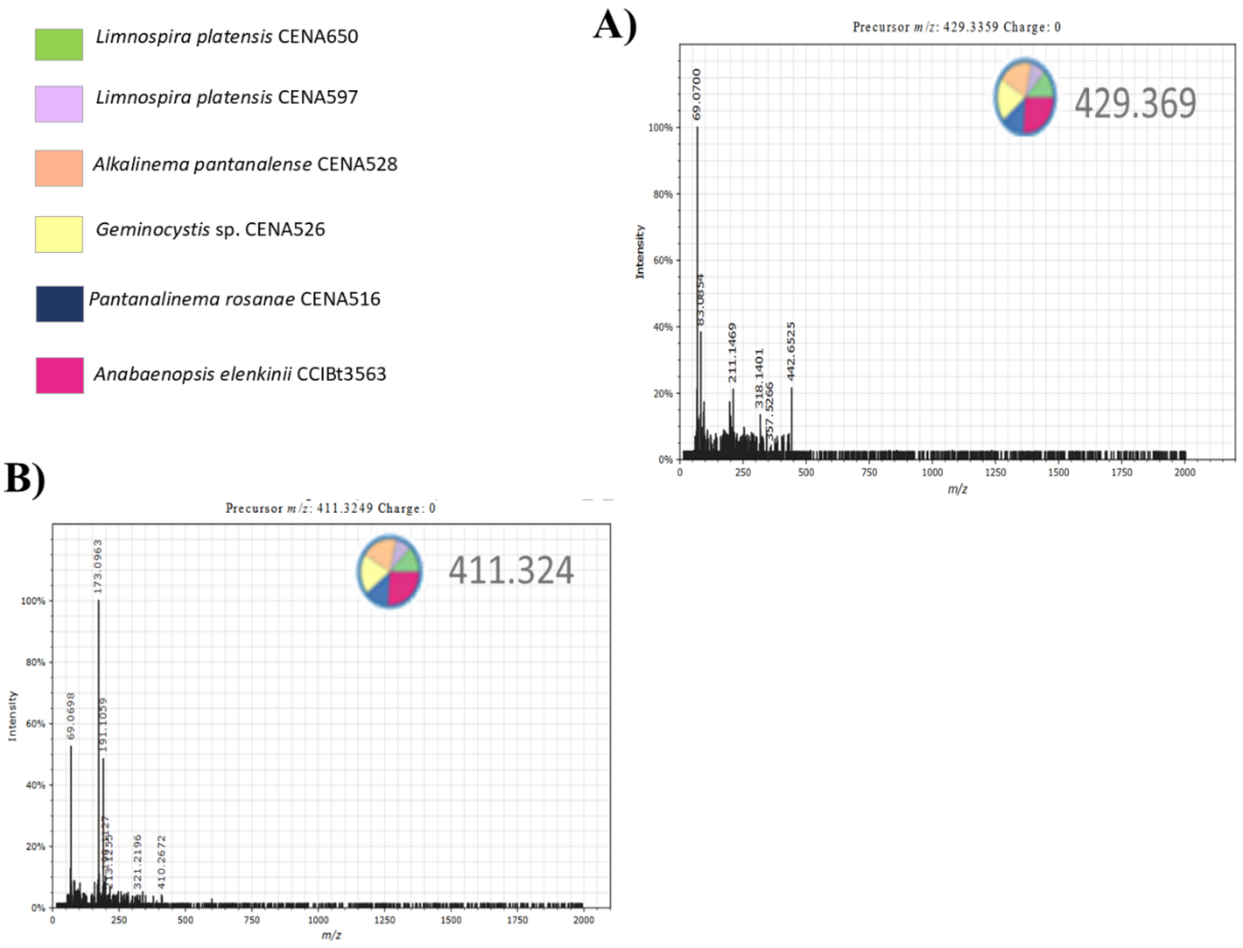

Figure S15. Ion product mass spectra of protonated terpenoids from cyanobacterial strains isolated from the Pantanal, Brazil. **A)** 429.369; **B)** 411.324. Arrows indicate the presence of fragments corresponding to the ionization of the molecular ion the same terpene. Nodes indicate the strains in which the compounds were detected.
