# Supplementary material for: Genetic and biochemical diversity of terpene biosynthesis in cyanobacterial strains from tropical soda lakes
Source: Front Microbiol. 2025 Jul 4;16:1582103. doi: 10.3389/fmicb.2025.1582103 (PMC12271159; doi:10.3389/fmicb.2025.1582103)
Supplement: Supplementary file 1 [file Supplementary_file_1.zip › Table S1.PDF]

Table S1. Characteristics of the cyanobacterial genomes used in the study, from saline-alkaline lakes of the Pantanal, sub-region of Nhecolândia, MS, Brazil.

Strain / Accesion nummber	Origin	Sequencing platform	Contigs	Total length	Largest contig	GC (%)	N50	Completeness	Contamination
Alkalinema pantanalense CENA 528 / JBLZFX000000000	Salina Preta, Centenário Farm, Aquidauana - 19°26'56.0"S, 56°07'54.8"W	Illumina Hiseq/PacBio HiFi	229	6,495,682	171,078	49.56	54,383	99.29	0.24
Geminocystis sp. CENA 526 / JBLZFY000000000	Centenário Salina, Centenário Farm, Aquidauana - 19°26'24"S, 56°05'58"W	Illumina Hiseq	320	4,843,400	164,417	34.36	35,250	99.78	0.66
Limnospira platensis CENA 597 / CP185278	Salina Grande, Centenário Farm, Aquidauana - 19°27'3.13"S, 56°7'42.19"W	Illumina Hiseq/PacBio HiFi	1	7,124,575	-	44.10	-	98.47	8.19
Limnospira platensis CENA 650 / JBLZFW000000000	Lake 08SR, São Roque Farm, Aquidauana - 19°22’ 47.2” S, 56°18’ 51.6” W	Illumina Hiseq	28	7,193,576	1,116,598	44.13	523,178	99,78	1,27
Pantanalinema rosanae CENA 516 / JBLZFZ000000000	Salina Verde, Centenário Farm, Aquidauana - 19°28'13"S, 56°03'22"W	Illumina Hiseq/PacBio HiFi	256	7,780,651	306,798	49.57	92,640	99.53	4.93
Anabaenopsis elenkinii CCIBt3563 / CP063311	Salina da Reserva, Nhumirim Farm, Corumbá - 18°57'35"S, 56°37'18"W	Illumina Hiseq	1	4,495,068	-	41.61	-	98.07	1.57
