# Supplementary material for: Genetic and biochemical diversity of terpene biosynthesis in cyanobacterial strains from tropical soda lakes
Source: Front Microbiol. 2025 Jul 4;16:1582103. doi: 10.3389/fmicb.2025.1582103 (PMC12271159; doi:10.3389/fmicb.2025.1582103)
Supplement: Supplementary file 1 [file Supplementary_file_1.zip › Table S2.PDF]

Table S2. Genes of terpene biosynthesis pathway annotated by BlastKoala, biosynthetic genes cluster (BCGs) predicted by AntiSMASH and compounds annotated in the metabolomics analysis in each strain isolated from Pantanal, Brazil.

Sample	Terpene Biosynthesis Pathway	Gene name/ Substrate=>Product [EC numbers]	BGCs Predicted by AntiSMASH	Principal Compounds Detected by LC-MS/MS (positive mode)	Exclusive and abundant compounds Detected by LC-MS/MS (positive mode)
Anabaenopsis elenkinii **CCIBt3563** Salina da Reserva, Nhumirim Farm, Corumbá	**Tetraterpene carotenoid**	**crtB - 15-cis-phytoene synthase** (2 Geranylgeranyl diphosphate => Phytoene [2.5.1.32]); **crtP - 15-cis-phytoene desaturase** (Phytoene => 9,15,9'-tricis-zeta-carotene [1.3.5.5]); **crtQ - zeta-carotene desaturase** (9,9'-dicis-zeta-carotene => 7,9,7',9'-tetracis-lycopene [1.3.5.6]); **crtH - prolycopene isomerase** (7,9,7',9'-tetracis-lycopene => Lycopene [5.2.1.13]); **CruA/P - lycopene cyclase**(Lycopene => beta-carotene [1.14.13.-]); **crtU - carotenoid phi/chi -ring synthase** (gamma-Carotene => Chlorobactene, beta-Carotene => beta-Isorenieratene, beta-Carotene => Isorenieratene, beta-Isorenieratene => Isorenieratene [1.3.99.39]; **crtR - beta-carotene hydroxylase** ((2'S)-Deoxymyxol 2'-(2,4-di-O-methyl-alpha-L-fucoside) => (3R,2'S)-Myxol 2'-(2,4-di-O-methyl-alpha-L-fucoside), (2'S)-Deoxymyxol 2'-alpha-L-fucoside => (3R,2'S)-Myxol 2'-alpha-L-fucoside, beta-carotene => beta-Cryptoxanthin, beta-Cryptoxanthin => Zeaxanthin, Echinenone => 3-Hydroxyechinenone [1.14.13.-])	Phytoene synthase (crtB)	β-Carotene (*m/z* 536,432), Hydroxycarotenoid (*m/z* 500,356), Echinenone (*m/z* 550,412), Zeaxanthin (*m/z*: 568,424)	**Exclusive:** Canthaxanthin - (*m/z* 565,399), myxoxanthophyll - (*m/z* 746,472), β-Apo-2'-carotenal - (*m/z* 509,507) **Abundant:** β-Carotene (*m/z* 565.562), Canthaxanthin (*m/z* 567.416) and β-Cryptoxanthin (567,416
	**Ubiquinone**	Ubiquinol synthesis: **ubiA - 4-hydroxybenzoate polyprenyltransferase** (Polyprenyl diphosphate + 4-hydroxybenzoate => 4-hydroxy-3-polyprenylbenzoate [2.5.1.39]); Vitamin K cycle: **menA - 2-carboxy-1,4-naphthoquinone phytyltransferase** (Phytyl diphosphate + 2-carboxy-1,4-naphthoquinone => Demethylphylloquinone [2.5.1.130]); alpha-Tocopherol (Vitamin E): **hpt - homogentisate phytyltransferase** (phytyl diphosphate + homogentisate => 2-methyl-6-phytylbenzene-1,4-diol [2.5.1.115]); alpha-Tocotrienol (Vitamin E): **hggt - homogentisate geranylgeranyltransferase** (geranylgeranyl diphosphate + homogentisate => 6-geranylgeranyl-2-methylbenzene-1,4-diol [2.5.1.116])		α-tocopherol_12',13'-Didehydro (*m/z* 429,369) α-tocotrienol (*m/z* 411,325)	
Pantanalinema rosanae **CENA516** Salina Verde, Centenário Farm, Aquidauana	**Tetraterpene carotenoid**	**crtB - 15-cis-phytoene synthase** (2 Geranylgeranyl diphosphate => Phytoene [2.5.1.32]); **crtP - 15-cis-phytoene desaturase** (Phytoene => 9,15,9'-tricis-zeta-carotene [1.3.5.5]); **crtQ - zeta-carotene desaturase** (9,9'-dicis-zeta-carotene => 7,9,7',9'-tetracis-lycopene [1.3.5.6]); **crtH - prolycopene isomerase** (7,9,7',9'-tetracis-lycopene => Lycopene [5.2.1.13]); **CruA/P - lycopene cyclase**(Lycopene => beta-carotene [1.14.13.-]); **crtR - beta-carotene hydroxylase** ((2'S)-Deoxymyxol 2'-(2,4-di-O-methyl-alpha-L-fucoside) => (3R,2'S)-Myxol 2'-(2,4-di-O-methyl-alpha-L-fucoside), (2'S)-Deoxymyxol 2'-alpha-L-fucoside => (3R,2'S)-Myxol 2'-alpha-L-fucoside, beta-carotene => beta-Cryptoxanthin, beta-Cryptoxanthin => Zeaxanthin, Echinenone => 3-Hydroxyechinenone [1.14.13.-]); **crtS - beta-carotene 4-ketolase** (beta-Carotene => Echinenone, Echinenone => Canthaxanthin, (3R,2'S)-Myxol 2'-alpha-L-fucoside => (3S,2'S)-4-Ketomyxol 2'-alpha-L-fucoside, beta-Cryptoxanthin => Hydroxyechinenone, 3'-Hydroxyechinenone => Phoenicoxanthin [1.14.99.63], **crtW - zeaxanthin 4-ketolase** (Zeaxanthin => Adonixanthin, Adonixanthin => Astaxanthin [1.14.99.64]; **crtX - zeaxanthin glucosyltransferase** (Zeaxanthin => Zeaxanthin diglucoside [2.4.1.276])	Phytoene synthase (crtB)	β-Carotene (*m/z:* 536,432), Hydroxycarotenoids (*m/z*: 500,356), Echinenone (*m/z*: 550,412, Zeaxanthin (*m/z*: 568,424)	**Exclusive**: Tetradehydro-2,2,diketo-beta-carotene (*m/z* 561,487) and apocarotenoid (*m/z* 608,521) **Abundant**: β-Carotene (*m/z* 536.432), Echinenone (*m/z* 565.562) and Zeaxantina (*m/z* 568,424)
	**Ubiquinone**	Ubiquinol synthesis: **ubiA - 4-hydroxybenzoate polyprenyltransferase** (Polyprenyl diphosphate + 4-hydroxybenzoate => 4-hydroxy-3-polyprenylbenzoate [2.5.1.39]); Vitamin K cycle: **menA - 2-carboxy-1,4-naphthoquinone phytyltransferase** (Phytyl diphosphate + 2-carboxy-1,4-naphthoquinone => Demethylphylloquinone [2.5.1.130]); alpha-Tocopherol (Vitamin E): **hpt - homogentisate phytyltransferase** (phytyl diphosphate + homogentisate => 2-methyl-6-phytylbenzene-1,4-diol [2.5.1.115]); alpha-Tocotrienol (Vitamin E): **hggt - homogentisate geranylgeranyltransferase** (geranylgeranyl diphosphate + homogentisate => 6-geranylgeranyl-2-methylbenzene-1,4-diol [2.5.1.116])		α-tocopherol_12',13'-Didehydro (*m/z* 429,369) α-tocotrienol (*m/z* 411,325)	
	**Triterpenoid**	**FDFT1 - farnesyl-diphosphate farnesyltransferase** (2 (2E,6E)-farnesyl diphosphate => Squalene [2.5.1.21]; **shc - squalene-hopene cyclase** (Hopan-22-ol => Squalene [4.2.1.129], Squalene => hop-22(29)-ene [5.4.99.17])	scalene hopene cyclase (sqhC)		
Geminocystis **sp. CENA526** Centenário Salina, Centenário Farm, Aquidauana	**Tetraterpene carotenoid**	**crtB - 15-cis-phytoene synthase** (2 Geranylgeranyl diphosphate => Phytoene [2.5.1.32]); **crtP - 15-cis-phytoene desaturase** (Phytoene => 9,15,9'-tricis-zeta-carotene [1.3.5.5]); **crtQ - zeta-carotene desaturase** (9,9'-dicis-zeta-carotene => 7,9,7',9'-tetracis-lycopene [1.3.5.6]); **crtH - prolycopene isomerase** (7,9,7',9'-tetracis-lycopene => Lycopene [5.2.1.13]); **CruA/P - lycopene cyclase**(Lycopene => beta-carotene [1.14.13.-]); **crtU - carotenoid phi/chi -ring synthase** (gamma-Carotene => Chlorobactene, beta-Carotene => beta-Isorenieratene, beta-Carotene => Isorenieratene, beta-Isorenieratene => Isorenieratene [1.3.99.39]; **crtR - beta-carotene hydroxylase** ((2'S)-Deoxymyxol 2'-(2,4-di-O-methyl-alpha-L-fucoside) => (3R,2'S)-Myxol 2'-(2,4-di-O-methyl-alpha-L-fucoside), (2'S)-Deoxymyxol 2'-alpha-L-fucoside => (3R,2'S)-Myxol 2'-alpha-L-fucoside, beta-carotene => beta-Cryptoxanthin, beta-Cryptoxanthin => Zeaxanthin, Echinenone => 3-Hydroxyechinenone [1.14.13.-]); **crtS - beta-carotene 4-ketolase** (beta-Carotene => Echinenone, Echinenone => Canthaxanthin, (3R,2'S)-Myxol 2'-alpha-L-fucoside => (3S,2'S)-4-Ketomyxol 2'-alpha-L-fucoside, beta-Cryptoxanthin => Hydroxyechinenone, 3'-Hydroxyechinenone => Phoenicoxanthin [1.14.99.63], **crtW - zeaxanthin 4-ketolase** (Zeaxanthin => Adonixanthin, Adonixanthin => Astaxanthin [1.14.99.64]	Phytoene synthase (crtB)	β-Carotene (*m/z:* 536,432), Hydroxycarotenoids (*m/z*: 500,356), Echinenone (*m/z*: 550,412, Zeaxanthin (*m/z*: 568,424)	**Exclusive:** Apocarotenoid glycosides - (*m/z*: 580.489, 585.426, 594.502, 620.517, 622.495, 624.495, 638.531, 664.555, 668.539, 694.554) and Phoenicoxanthin (*m/z*: 581.429). **Abundant:** α- Cryptoxanthin *m/z* 552.427, Zeaxantina *m/z* 568,424 and Echinenone m/z 565,562
	**Ubiquinone**	Ubiquinol synthesis: **ubiA - 4-hydroxybenzoate polyprenyltransferase** (Polyprenyl diphosphate + 4-hydroxybenzoate => 4-hydroxy-3-polyprenylbenzoate [2.5.1.39]); Vitamin K cycle: **menA - 2-carboxy-1,4-naphthoquinone phytyltransferase** (Phytyl diphosphate + 2-carboxy-1,4-naphthoquinone => Demethylphylloquinone [2.5.1.130])		α-tocopherol_12',13'-Didehydro (*m/z* 429,369) α-tocotrienol (*m/z* 411,325)	
	**Triterpenoid**	**FDFT1 - farnesyl-diphosphate farnesyltransferase** (2 (2E,6E)-farnesyl diphosphate => Squalene [2.5.1.21]; **shc - squalene-hopene cyclase** (Hopan-22-ol => Squalene [4.2.1.129], Squalene => hop-22(29)-ene [5.4.99.17])	scalene hopene cyclase (sqhC)	35-O-β-6 amino-6-deoxyglucopyranosyl, Bacteriohopanetetrol	**Exclusive:** 35-O-β-6-amino-6-deoxyglucopyranosyl, and Bacteriohopanetetrol

Table S2 continuation. Genes of terpene biosynthesis pathway annotated by BlastKoala, biosynthetic genes cluster (BCGs) predicted by AntiSMASH and compounds annotated in the metabolomics analysis in each strain isolated from Pantanal, Brazil.

**Sample**	**Terpene Biosynthesis Pathway**	**Gene name/ Substrate-product [EC numbers]**	**BGCs Predicted by AntiSMASH**	**Principal Compounds Detected by LC-MS/MS (positive mode)**	**Exclusive and abundant compounds Detected by LC-MS/MS (positive mode)**
***Alkalinema pantanalense* CENA528** Salina Preta, Centenário Farm, Aquidauana	**Tetraterpene carotenoid**	**crtB - 15-cis-phytoene synthase** (2 Geranylgeranyl diphosphate => Phytoene [2.5.1.32]); **crtP - 15-cis-phytoene desaturase** (Phytoene => 9,15,9'-tricis-zeta-carotene [1.3.5.5]); **crtQ - zeta-carotene desaturase** (9,9'-dicis-zeta-carotene => 7,9,7',9'-tetracis-lycopene [1.3.5.6]); **crtH - prolycopene isomerase** (7,9,7',9'-tetracis-lycopene => Lycopene [5.2.1.13]); **CruA/P - lycopene cyclase**(Lycopene => beta-carotene [1.14.13.-]); **crtR - beta-carotene hydroxylase** ((2'S)-Deoxymyxol 2'-(2,4-di-O-methyl-alpha-L-fucoside) => (3R,2'S)-Myxol 2'-(2,4-di-O-methyl-alpha-L-fucoside), (2'S)-Deoxymyxol 2'-alpha-L-fucoside => (3R,2'S)-Myxol 2'-alpha-L-fucoside, beta-carotene => beta-Cryptoxanthin, beta-Cryptoxanthin => Zeaxanthin, Echinenone => 3-Hydroxyechinenone [1.14.13.-]); **crtS - beta-carotene 4-ketolase** (beta-Carotene => Echinenone, Echinenone => Canthaxanthin, (3R,2'S)-Myxol 2'-alpha-L-fucoside => (3S,2'S)-4-Ketomyxol 2'-alpha-L-fucoside, beta-Cryptoxanthin => Hydroxyechinenone, 3'-Hydroxyechinenone => Phoenicoxanthin [1.14.99.63], **crtW - zeaxanthin 4-ketolase** (Zeaxanthin => Adonixanthin, Adonixanthin => Astaxanthin [1.14.99.64]; **crtX - zeaxanthin glucosyltransferase** (Zeaxanthin => Zeaxanthin diglucoside [2.4.1.276])	Phytoene synthase (crtB)	β-Carotene (*m/z:* 536,432), Hydroxycarotenoids (*m/z*: 500,356), Echinenone (*m/z*: 550,412, Zeaxanthin (*m/z*: 568,424)	**Exclusive**: Ketohydroxylycopene (*m/z* 566,547), Glycosyl-4,4'-diaponeurosporenoate (*m/z* 594,577), 1,2-Dihydrolycopene (*m/z* 538,515) **Abundant:** 3-Hydroxyechinenone (*m/z* 566.547), β-Cryptoxanthin (*m/z* 567.416) and β-Carotene (*m/z* 536.432).
	**Ubiquinone**	<u>Ubiquinol synthesis</u>: **ubiA - 4-hydroxybenzoate polyprenyltransferase** (Polyprenyl diphosphate + 4-hydroxybenzoate => 4-hydroxy-3-polyprenylbenzoate [2.5.1.39]); <u>Vitamin K cycle</u>: **menA - 2-carboxy-1,4-naphthoquinone phytyltransferase** (Phytyl diphosphate + 2-carboxy-1,4-naphthoquinone => Demethylphylloquinone [2.5.1.130])		α-tocopherol_12',13'-Didehydro (*m/z* 429,369) α-tocotrienol (*m/z* 411,325)	
	**Triterpenoid**	**FDFT1 - farnesyl-diphosphate farnesyltransferase** (2 (2E,6E)-farnesyl diphosphate => Squalene [2.5.1.21]; **shc - squalene-hopene cyclase** (Hopan-22-ol => Squalene [4.2.1.129], Squalene => hop-22(29)-ene [5.4.99.17])	scalene hopene cyclase (sqhC)		
***Limnospira platensis* CENA597** Salina Grande, Centenário Farm, Aquidauana	**Tetraterpene carotenoid**	**crtB - 15-cis-phytoene synthase** (2 Geranylgeranyl diphosphate => Phytoene [2.5.1.32]); **crtP - 15-cis-phytoene desaturase** (Phytoene => 9,15,9'-tricis-zeta-carotene [1.3.5.5]); **crtQ - zeta-carotene desaturase** (9,9'-dicis-zeta-carotene => 7,9,7',9'-tetracis-lycopene [1.3.5.6]); **crtH - prolycopene isomerase** (7,9,7',9'-tetracis-lycopene => Lycopene [5.2.1.13]); **CruA/P - lycopene cyclase**(Lycopene => beta-carotene [1.14.13.-]); **crtU - carotenoid phi/chi -ring synthase** (gamma-Carotene => Chlorobactene, beta-Carotene => beta-Isorenieratene, beta-Carotene => Isorenieratene, beta-Isorenieratene => Isorenieratene [1.3.99.39]; **crtR - beta-carotene hydroxylase** ((2'S)-Deoxymyxol 2'-(2,4-di-O-methyl-alpha-L-fucoside) => (3R,2'S)-Myxol 2'-(2,4-di-O-methyl-alpha-L-fucoside), (2'S)-Deoxymyxol 2'-alpha-L-fucoside => (3R,2'S)-Myxol 2'-alpha-L-fucoside, beta-carotene => beta-Cryptoxanthin, beta-Cryptoxanthin => Zeaxanthin, Echinenone => 3-Hydroxyechinenone [1.14.13.-])	Phytoene synthase (crtB)	β-Carotene (*m/z:* 536,432), Hydroxycarotenoids (*m/z*: 500,356), Echinenone (*m/z*: 550,412, Zeaxanthin (*m/z*: 568,424), 3,3'-Ditetrahydropyranosyloxyisorenieratene (*m/z* 729,469)	**Exclusive:** Adonixanthin (*m/z* 582,40), Myxoxanthophyll (*m/z* 703,448) **Abundant:** β-Carotene (*m/z* 565.562), além de Diatoxanthin (*m/z* 567.416) and β-Cryptoxanthin (*m/z* 553.435).
	**Ubiquinone**	<u>Ubiquinol synthesis</u>: **ubiA - 4-hydroxybenzoate polyprenyltransferase** (Polyprenyl diphosphate + 4-hydroxybenzoate => 4-hydroxy-3-polyprenylbenzoate [2.5.1.39]); <u>Vitamin K cycle</u>: **menA - 2-carboxy-1,4-naphthoquinone phytyltransferase** (Phytyl diphosphate + 2-carboxy-1,4-naphthoquinone => Demethylphylloquinone [2.5.1.130]); <u>alpha-Tocopherol</u> (Vitamin E): **hpt - homogentisate phytyltransferase** (phytyl diphosphate + homogentisate => 2-methyl-6-phytylbenzene-1,4-diol [2.5.1.115]); <u>alpha-Tocotrienol</u> (Vitamin E): **hggt - homogentisate geranylgeranyltransferase** (geranylgeranyl diphosphate + homogentisate => 6-geranylgeranyl-2-methylbenzene-1,4-diol [2.5.1.116])		α-tocopherol_12',13'-Didehydro (*m/z* 429,369) α-tocotrienol (*m/z* 411,325)	
***Limnospira platensis* CENA650** 08SR Lake, São Roque Farm, Aquidauana	**Tetraterpene carotenoid**	**crtB - 15-cis-phytoene synthase** (2 Geranylgeranyl diphosphate => Phytoene [2.5.1.32]); **crtP - 15-cis-phytoene desaturase** (Phytoene => 9,15,9'-tricis-zeta-carotene [1.3.5.5]); **crtQ - zeta-carotene desaturase** (9,9'-dicis-zeta-carotene => 7,9,7',9'-tetracis-lycopene [1.3.5.6]); **crtH - prolycopene isomerase** (7,9,7',9'-tetracis-lycopene => Lycopene [5.2.1.13]); **CruA/P - lycopene cyclase**(Lycopene => beta-carotene [1.14.13.-]); **crtU - carotenoid phi/chi -ring synthase** (gamma-Carotene => Chlorobactene, beta-Carotene => beta-Isorenieratene, beta-Carotene => Isorenieratene, beta-Isorenieratene => Isorenieratene [1.3.99.39]; **crtR - beta-carotene hydroxylase** ((2'S)-Deoxymyxol 2'-(2,4-di-O-methyl-alpha-L-fucoside) => (3R,2'S)-Myxol 2'-(2,4-di-O-methyl-alpha-L-fucoside), (2'S)-Deoxymyxol 2'-alpha-L-fucoside => (3R,2'S)-Myxol 2'-alpha-L-fucoside, beta-carotene => beta-Cryptoxanthin, beta-Cryptoxanthin => Zeaxanthin, Echinenone => 3-Hydroxyechinenone [1.14.13.-])	Phytoene synthase (crtB)	β-Carotene (*m/z:* 536,432), Echinenone (*m/z*: 550,412, Zeaxanthin (*m/z*: 568,424), 3,3'-Ditetrahydropyranosyloxyisorenieratene (*m/z* 729,469)	**Exclusive**: Xanthophyll (*m/z* 542,454), Tetrahydrolycopene (*m/z* 540,438) **Abundant:** β-Carotene (*m/z* 565.562), β-Cryptoxanthin (*m/z* 553.435 and 567.416)
	**Ubiquinone**	<u>Ubiquinol synthesis</u>: **ubiA - 4-hydroxybenzoate polyprenyltransferase** (Polyprenyl diphosphate + 4-hydroxybenzoate => 4-hydroxy-3-polyprenylbenzoate [2.5.1.39]); <u>Vitamin K cycle</u>: **menA - 2-carboxy-1,4-naphthoquinone phytyltransferase** (Phytyl diphosphate + 2-carboxy-1,4-naphthoquinone => Demethylphylloquinone [2.5.1.130]); <u>alpha-Tocopherol</u> (Vitamin E): **hpt - homogentisate phytyltransferase** (phytyl diphosphate + homogentisate => 2-methyl-6-phytylbenzene-1,4-diol [2.5.1.115]); <u>alpha-Tocotrienol</u> (Vitamin E): **hggt - homogentisate geranylgeranyltransferase** (geranylgeranyl diphosphate + homogentisate => 6-geranylgeranyl-2-methylbenzene-1,4-diol [2.5.1.116])		α-tocopherol_12',13'-Didehydro (*m/z* 429,369) α-tocotrienol (*m/z* 411,325)	
